# Molecular Dynamic Simulations Reveal that Water-Soluble QTY-Variants of Glutamate Transporters EAA1, EAA2 and EAA3 Retain the Conformational Characteristics of Native Transporters

**DOI:** 10.1007/s11095-024-03769-0

**Published:** 2024-09-25

**Authors:** Alper Karagöl, Taner Karagöl, Shuguang Zhang

**Affiliations:** 1 Istanbul University Istanbul Medical Faculty, Istanbul, Turkey; 2 Laboratory of Molecular Architecture, Media Lab, Massachusetts Institute of Technology, Massachusetts Avenue, Cambridge, MA 02139 USA

**Keywords:** convert hydrophobic helix to hydrophilic helix, lipid interactions, protein 3D structural predictions, QTY code, water-soluble integral membrane proteins

## Abstract

**Objective:**

Glutamate transporters play a crucial role in neurotransmitter homeostasis, but studying their structure and function is challenging due to their membrane-bound nature. This study aims to investigate whether water-soluble QTY-variants of glutamate transporters EAA1, EAA2 and EAA3 retain the conformational characteristics and dynamics of native membrane-bound transporters.

**Methods:**

Molecular dynamics simulations and comparative genomics were used to analyze the structural dynamics of both native transporters and their QTY-variants. Native transporters were simulated in lipid bilayers, while QTY-variants were simulated in aqueous solution. Lipid distortions, relative solvent accessibilities, and conformational changes were examined. Evolutionary conservation profiles were correlated with structural dynamics. Statistical analyses included multivariate analysis to account for confounding variables.

**Results:**

QTY-variants exhibited similar residue-wise conformational dynamics to their native counterparts, with correlation coefficients of 0.73 and 0.56 for EAA1 and EAA3, respectively (*p* < 0.001). Hydrophobic interactions of native helices correlated with water interactions of QTY- helices (rs = 0.4753, *p* < 0.001 for EAA1). QTY-variants underwent conformational changes resembling the outward-to-inward transition of native transporters.

**Conclusions:**

Water-soluble QTY-variants retain key structural properties of native glutamate transporters and mimic aspects of native lipid interactions, including conformational flexibility. This research provides valuable insights into the conformational changes and molecular mechanisms of glutamate transport, potentially offering a new approach for studying membrane protein dynamics and drug interactions.

**Supplementary information:**

The online version contains supplementary material available at 10.1007/s11095-024-03769-0.

## Introduction

Excitatory amino acid transporters (EAATs) are the main members of the glutamate transporter family [1–5], a class of membrane proteins playing a critical role in the central nervous system by removing glutamate from the synapse [1, 2]. This reuptake process helps to prevent excitotoxicity, as cell death could occur in the case of excessive glutamate concentrations [1–3]. EAATs function through specific structural conformations: namely inward-facing structure (IFS) and outward-facing structure (OFS) [2, 4, 6, 7]. During the transport cycle, the conversion of structural states is facilitated by the movement of the transmembrane domains, which reorient the protein core from facing the extracellular space (OFS) to the intracellular space (IFS), and reversible stochastic changes to the intermediate state occur [2, 4, 7]. Structural conformations emerge as a crucial aspect of the physiological glutamate transport cycle, as various molecules have been identified that disturb the conformation cycles and also hinder the transport of glutamate [3, 5, 6].

Despite their established roles, structural information on the glutamate transporters is still limited and many aspects of their molecular mechanisms have remained unknown [2]. This outcome is mainly due to the membranous nature of the transporters, as studies involving them are a daunting task for researchers [8]. To address this, we presented a method for designing water-soluble domains, instead of using detergents [9, 10]. The 1.5Å electron density maps show remarkable structural similarities between leucine (L) vs glutamine (Q); isoleucine (I), valine (V) vs threonine (T); and phenylalanine (F) vs tyrosine (Y) [9–11]. These characteristics lead to the implementation of the QTY-code (and reverse QTY-code) to modulate hydrophilicity of transmembrane domains [10–12]. Previous applications of the QTY code to chemokine and cytokine receptors have demonstrated that the resulting water-soluble variants maintain their predicted characteristics and ligand-binding activities [10–15]. Following the release of AlphaFold2 in 2021, we achieved rapid and more reliable QTY-variant protein structure predictions [16–20]. These predictions have shown that QTY-variants of glutamate and vesicular glutamate transporters exhibit structural similarities to their native membrane-bound counterparts [19, 20].

Our study differs from these structural analyses by considering the dynamical nature of the transport cycle, hypothesizing that water-soluble QTY helices can mimic aspects of native hydrophobic helix interactions. In the context of designed variants, identifying conformational spaces is a challenging process. Hence, we employed a comparative approach considering both water-soluble variants and native transporters. Subsequently, we statistically analyzed the data generated from molecular dynamic simulations and homology analysis. The evolutionary conservation of glutamate transporters across species suggests their adherence to an evolutionary stable strategy, making them ideal targets for evolutionary studies [4, 19, 21]. Previous studies identified the natural and evolutionary presence of the QTY-like mutations in glutamate transporters [19]. Reverse mutations of Q- > L, T- > I, Y- > F were already observed, indicating natural evolutionary reversibility [19, 20]. The existence of multiple structural conformations of EAATs may also be subject to evolutionary trade-offs.

In this study, we present a comprehensive analysis of water-soluble QTY-variants of the glutamate transporter subfamily. Meanwhile, we explain the impact of evolutionary diversity on the structural conformations and their environmental interactions. offering a framework supported by molecular dynamics simulations. Hereby proposed characteristics of water-soluble QTY-variants introduce a new layer to our understanding of protein dynamics, evolution of lipid interactions, and identifies potential therapeutic targets.

## Results and Discussions

### Psychochemical and Evolutionary Properties of the QTY-Variants

The topological visualizations and predicted sequence features of EAATs indicated that each transporter has an 8 transmembrane (TM) architecture (Figure S1). The native structures have a high hydrophobicity content, particularly in their transmembrane alpha-helical segments, causing them to be insoluble in water and necessitating the use of surfactants for isolation. Meanwhile, QTY-variants have soluble characteristics and do not have TM domains as predicted by sequence-based analysis (Figure S2, Figure S3) [19]. To achieve the hydrophilic presentation, the QTY code presents significant substitutions, particularly in the transmembrane helices, ranging from 41 to 54%, as calculated in our previous paper [19]. Despite the high substitution rate, the difference in molecular weight between the native and QTY-variants is minimal, in the range of a few hundred Daltons (Da) (Supplementary Table S1). Additionally, the QTY substitutions do not introduce any charged residues into the protein, thus resulting in minimal changes of isoelectric points (pIs), which could lead to non-specific interactions if changed [19]. Furthermore, in our previous analysis, the experimental structures of native proteins and their AlphaFold2 predicted water-soluble QTY-variants were shown to superpose well, as demonstrated by the root mean square deviation (Supplementary Table S1, S2). By replacing the hydrophobic amino acids L, I, V, and F with hydrophilic ones (Q, T, Y), the hydrophobic surfaces were significantly reduced [19].

The QTY substitutions are also observed in nature and may have particular roles in physiology or diagnosis [19, 20]. Over 40 QTY base substitutions were previously identified in EAA1, EAA2 and EAA3, including those targeting the highly conserved helical residues [19]. Computational analysis indicates the QTY-variants were significantly less damaging compared to other substitutions, regardless of the occurrence in nature [19, 20]. Furthermore, previous evolutionary studies revealed a co-evolution pattern of QTY-code pairs in monoamine transporters, including transporters for dopamine and serotonin neurotransmitters, that follow similar conservation profiles with the glutamate transporter subfamily [19, 20]. The statistically significant correlation between the co-occurrence of QTY-code amino acids in natural sequences supports their evolutionary adaptation.

## Structural Variability of the EAA1, EAA2 and EAA3

The conformational dynamics of glutamate transporters are crucial for their function [2, 4, 6, 7]. One well-studied conformational change in glutamate transporters is the transition between outward-facing and inward-facing states [2, 4, 7]. In the outward-facing conformation, the transporter core is exposed to the extracellular environment; a series of subsequent structural changes result in the core being relatively inward-facing [2, 4, 7]. Prediction of the conformational states is a daunting task, since AlphaFold aims to predict the most likely protein structure based on the given input data and does not necessarily adhere to the Boltzmann form, the probabilistic occurrence seen in nature [8, 22]. On the other hand, AlphaFold2 predictions were in line with the experimentally derived conformations of glutamate transporters, with root mean square deviation (RMSD) values as close as 1Å compared to experimental outward structures derived from X-ray and cryo-EM (Supplementary Table S2).

Despite a high substitution rate in the transmembrane alpha-helices in the water-soluble QTY-variants, their structures remain similar to the native structures; demonstrated by the RMSD values [19]. The AlphaFold-predicted first-ranking QTY-variants have retained similar conformations as native models. The structural similarity between the native and QTY-variants was high, with RMSD values below 1Å (Supplementary Table S1). The initial structures are important in MD simulations; this correlation would make them better targets for further comparative studies [19].

In contrast to EAA1, EAA3 is mostly intracellular and has a rapid regulation of its membrane trafficking [1, 23]. Another significant feature of glutamate transporters is their cyclic assemblies consisting of three identical monomer units, inter-monomer contacts in the homotrimer are mediated by TM2, TM4, and TM5 [2, 24]. Interestingly, these motifs were found to be conserved (Figure S4). AlphaFold multimer predictions of the EAA1, EAA2 and EAA3 trimer conformations also correlated with their model ranking. Higher rankings were mostly outward-facing conformations, while ranks 4–5 tended to be inward-facing structures of the transporter (Supplementary Table S3).

### Analysis of Lipid Distortions and Relative Solvent Accessibilities

**Fig. 1:**
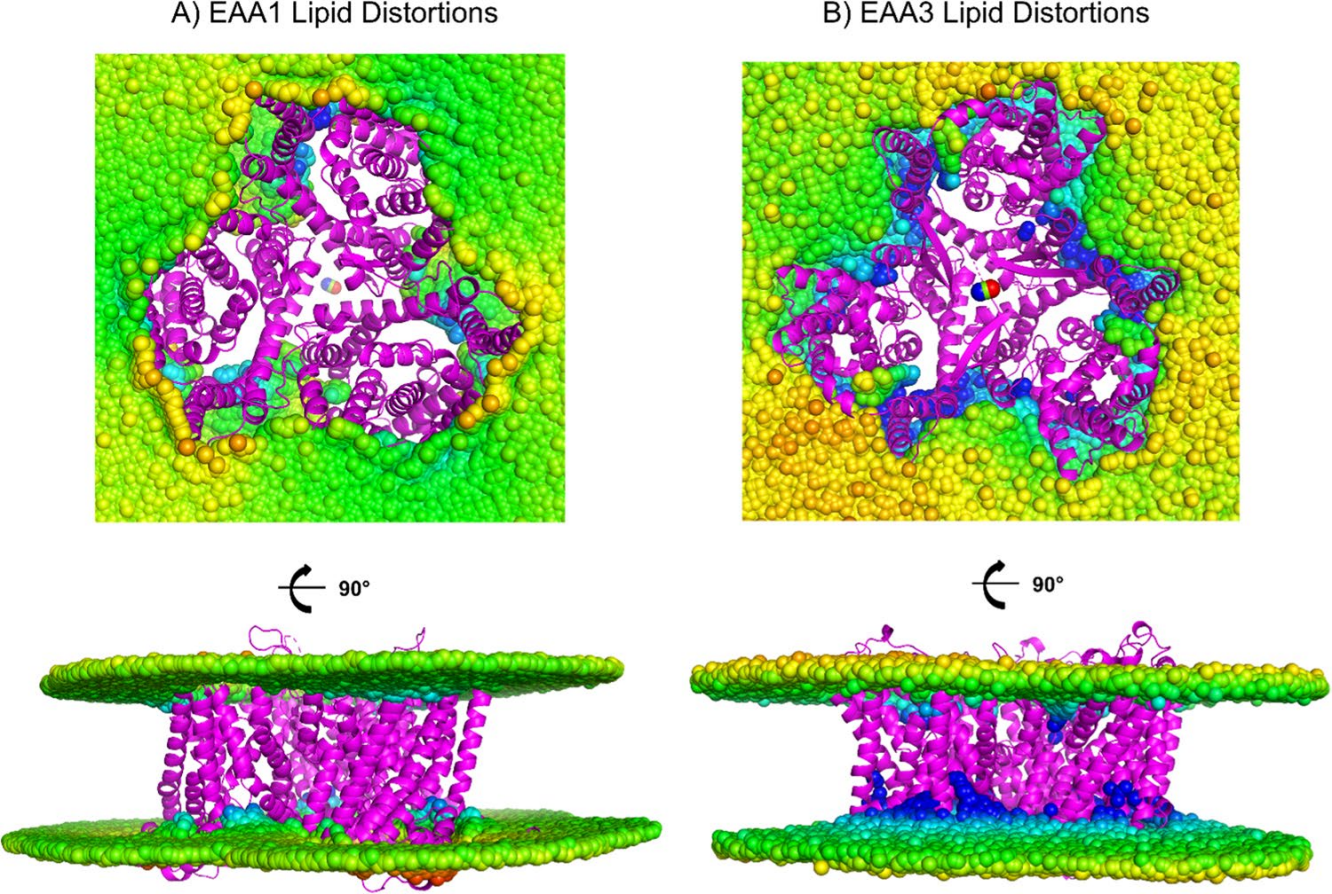
Lipid distortions of the experimental EAA1 and EAA3 homotrimer structures over 800 ns MD. Lipid distortions depict the mean surface profile over the concluding 800 ns of simulation. Blue indicates a thinning of the bilayer (compared to bulk thickness), while red indicates thickening. For clarity, the N- and C-termini and large loops, which are not resolved in experimental structures, were deleted.

Lipid distortions were analyzed from the data generated through 800ns MD simulations of experimental models of EAA1 (7NPW) and EAA3 (8CV2) (Fig. 1). Regarding the EAA1 bio-assembly, notable thinning occurred predominantly in the upper layer. Specifically, thinning of -1.71Å was observed in the upper leaflet, while thinning in the lower leaflet was measured at 0.71Å. At the point of maximum closure, the lipid heads of both the upper and lower leaflets approached within a distance of 28.1Å, deviating from the 38.8Å annular thickness (Figure S5). The lipid distortions were even more prominent for EAA3, with thinning still occurring predominantly in the upper layer. Thinning of -7.30Å was observed in the upper leaflet, while thinning in the lower leaflet was measured at -1.06Å. Notably, the lipid heads of the upper and lower leaflets approached within a distance of 15.6Å, from the 33.5Å annular thickness (Figure S6).

**Fig. 2:**
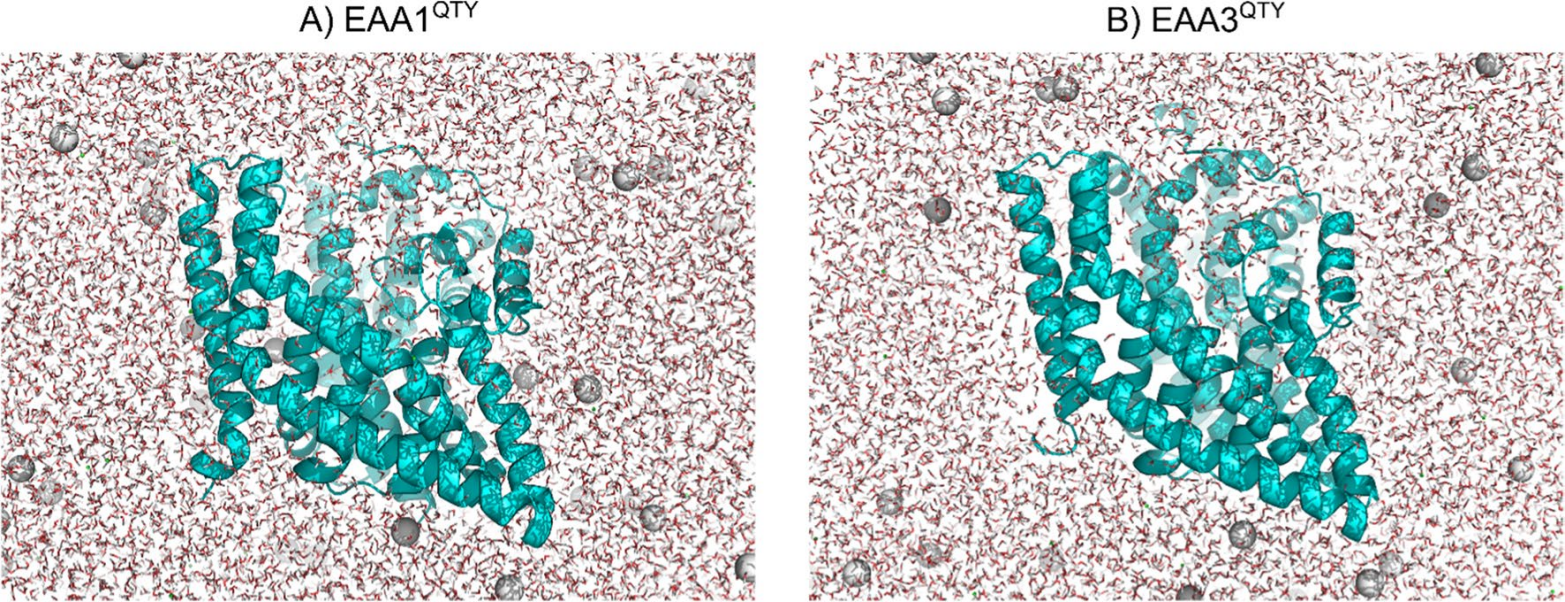
Water-soluble monomers of EAA1^QTY^ and EAA3^QTY^ in solution. The QTY-variants in solution with Monte-Carlo placed K^+^ CL^−^ ions (neutralizing, concentration = 0.15 M) (Methods). For clarity, the N- and C-termini and large loops, which are not resolved in experimental structures, were deleted.

The water soluble QTY-variants could not be in the lipid bilayer with their hydrophilic helices (Fig. 2). However, the solvent-accessible surface areas (SASA) of residues remained diverse: σ^2^ = 2429.8 (n = 416AA) and σ^2^ = 2389.8 (n = 407AA) for EAA1^QTY^ and EAA3^QTY^, respectively (Fig. 2). On the other hand, SASA is not sensitive to amino acid size [25]. Relative solvent accessibility (RSA) is calculated by removing other residues except its two adjacent neighbors. RSA is suitable for this study since it is sensitive to conformational changes [25]. Furthermore, it aligns more with the guiding principle of the QTY code, as it is derived from electron map similarities among chemically different amino acids [10]. The outer residues of TM helices and some inner core helices are closer to their maximum possible solvent-accessible surface area (Figure S7). This suggests that these regions are more exposed to the solvent environment than other regions of the protein.

An apparent positive correlation between the RSA of EAA1^QTY^ residues and lipid head contacts of native EAA1 residues was identified (rs = 0.4753, *p* < 0.001). The correlation was also notable with the solvent (water) and acyl contacts of native residues (rs = 0.347 and rs = 0.225, respectively). The colorations of EAA3^QTY^ were also prominent: rs = 0.3117 for lipid heads, rs = 0.301 for water contacts, and rs = 0.123 for acyl contacts. The reduced correlation with acyl tails for EAA3^QTY^ implies that while QTY helices can mimic aspects of the native lipid interactions (particularly with lipid heads and water), replicating interactions with the hydrophobic regions of lipids (acyl tails) might be more difficult compared to EAA1. These findings suggest that the water interactions with helices of QTY variants significantly correlated with the interactions between lipid heads and native transporters.

Hydrophobic acyl tails also seemed to be correlated with the surface accessibility profiles to a lesser extent in both EAATs. This is expected since water-soluble residues of native transporters were retained in QTY variants, the substitutions occurred in hydrophobic residues, and this may result in a confounding effect towards water contacts. Multivariate analysis was utilized to disentangle the effects of different confounding variables and isolate the specific impact of QTY modifications on protein-lipid interactions. After removing the confounding variables of water contact in native transporters, the correlation coefficient between acyl contacts and RSAs became more pronounced: rs = 0.4457 for EAA1^QTY^ and rs = 0.281 for EAA3^QTY^. The hydrophobic acyl interactions were found to be comparable to those of hydrophilic contacts in QTY variants (*p* < 0.001). In this case, water interactions with the hydrophilic QTY helices could be an important subsidy for the lipid interactions with hydrophobic helices in native transporters.

### Residue-wise RMSD Fluctuations and Experimental Structural States

Comparative analysis of the RMSD fluctuations indicates notable similarities between the native transporters in lipid bilayers and their water-soluble QTY-variants in solvent. In the case of EAA1, the strong correlation coefficient (rs = 0.73, *p* < 0.001) observed between the residue-wise RMSD values of the two systems suggests a significant degree of dynamic similarity, despite their disparate environments. For EAA2 and EAA3, the correlations were also significant (*p* < 0.001), though with slightly lower coefficients (rs = 0.39 and rs = 0.56, respectively). These results indicate that the residues that contributed to the conformational dynamics retained their roles in water-soluble QTY-variants, regardless of the effect of the lipid bilayer (Figure S8).

The most significant deviations occurred between residues 230 and 400, correlating with the transition to intermediate structures. The outward experimental models were almost identical to the QTY-variants (Table I). As the simulation progressed, the EAA3^QTY^ showed a dynamic change towards a more inward intermediate state. The partial RMSD value between the initial outward state of EAA3^QTY^ and intermediate experimental state of native EAA3 is 1.99Å, and after 100ns MD it is decreased to 1.74Å, indicating a closer alignment with the intermediate conformation and highlighting the dynamic regions within residues 230 and 400. This analysis indicates the interaction between the inner core with the outer helices such as TM5 is a major change for this transition. TM5 is also a member of the trimerization domain and contributes to homotrimer formation [2, 24]. Thus, cyclic bioassembly formation may have roles in conformational stability.

**Table I:** Conformations of EAA3^QTY^ and RMSD from Experimental CRYO-EM Obtained EAA3 Structural States

Name	Time step^1^	RMSD from the 8CV2 (outward state)^2^	RMSD from the 8CV3 (intermediate state)^3^
EAA3^QTY^	Initial (0nsMD)	1.137 Å	2.211 Å
	20nsMD	1.521 Å	2.398 Å
	40nsMD	1.691 Å	2.735 Å
	60nsMD	2.117 Å	2.682 Å
	80nsMD	2.227 Å	2.688 Å
	100nsMD	2.734 Å	2.342 Å

^1^The molecular dynamics simulation time point at which the conformation was observed, measured in nanoseconds (ns)

^2^Root Mean Square Deviation (RMSD) in Angstroms (Å) between the simulated EAA3^QTY^ structure and the experimentally determined outward state structure (PDB ID: 8CV2)

^3^Root Mean Square Deviation (RMSD) in Angstroms (Å) between the simulated EAA3QTY structure and the experimentally determined intermediate state structure (PDB ID: 8CV3)

### Dynamic Behaviors of the EAA1^QTY^, EAA2^QTY^, EAA3^QTY^ Structures in Water Solvent

Correlated with the diversity of native lipid distortion profiles, the QTY-variants also exhibited dynamic disparities amongst each transporter. Water-soluble QTY-variants of EAA2 and EAA3 were more flexible than EAA1 (Fig. 4). This result is correlated with the more profound membrane thinning profiles of EAA2 and EAA3. Lipid distortions around native EAA1 were much less pronounced than those around EAA3, suggesting a more stable interaction with the membrane in EAA1. Supporting this, EAA2^QTY^ and EAA3^QTY^ demonstrated greater versatility during the MD simulations, showing more substantial conformational changes. These rapid changes were not observed in membranous MD simulations of native EAA2 and EAA3 transporters, indicating that membrane lipids play stabilizing roles against the inward transition. This is consistent with previous analyses of the roles of lipid interactions on EAA3 conformational changes [7, 26]. On the other hand, the volatile residues of native transporters in the lipid bilayer were still in line with those of QTY-variants in water solvent, as evident in the correlation analysis of residue-wise RMSDs (Table II).

**Table II:** RMSD between Conformations of EAA1^QTY^, EAA2^QTY^ and EAA3^QTY^ with Native EAA1, EAA2, EAA3 through 100 ns MD Simulations

Name	Time step^1^	Native Deviation from the initial structure^2^	Deviation from the initial structure^3^
EAA1^QTY^	Initial	0 Å	0 Å
	20nsMD	1.54 Å	1.97 Å
	40nsMD	1.44 Å	1.89 Å
	60nsMD	1.53 Å	1.86 Å
	80nsMD	1.58 Å	2.08 Å
	100nsMD	1.96 Å	2.28 Å
	100nsMD (residue-wise correlation)	rs = 0.73, *p* < 0.001	
EAA2^QTY^	Initial	0 Å	0 Å
	20nsMD	1.45 Å	2.69 Å
	40nsMD	1.13 Å	2.89 Å
	60nsMD	1.33 Å	3.01 Å
	80nsMD	1.33 Å	3.47 Å
	100nsMD	1.61 Å	3.70 Å
	100nsMD (residue-wise correlation)	rs = 0.39, *p* < 0.001	
EAA3^QTY^	Initial	0 Å	0 Å
	20nsMD	1.27 Å	1.59 Å
	40nsMD	1.53 Å	1.97 Å
	60nsMD	1.24 Å	2.42 Å
	80nsMD	1.61 Å	3.05 Å
	100nsMD	1.46 Å	4.42 Å
	100nsMD (residue-wise correlation)	rs = 0.56, *p* < 0.001	

^1^The time point of the MD simulation at which the structure was analyzed

^2^The RMSD the native transporter from its initial state, measured in Angstroms (Å). It indicates how much the structure has changed from its initial conformation at each time point of the MD simulation

^3^The RMSD of the water-soluble QTY-variant from its initial state, measured in Angstroms (Å). It indicates how much the structure has changed from its initial conformation at each time point of the MD simulation

Within the lipid bilayer, native transporters interact with the hydrophobic tails of the lipid molecules, which can influence their conformational dynamics (Fig. 1). These interactions are crucial for maintaining the stability and functionality of the transporters within the membrane. On the other hand, the water-soluble QTY-variants experience interactions with water molecules (Fig. 2). These interactions are generally weaker than those within the lipid bilayer environment. The slight differences in RMSD between the native transporters and their water-soluble variants reflect the structural adaptability of these biomolecules to their respective environments. Despite differences in their surroundings, native transporters and their water-soluble variants share conserved structural elements essential for their function. These elements, such as transmembrane helices or flexible domains, maintain similar overall folds across native transporters and QTY-variants. Supporting this, normal mode analysis suggests strong similarities in eigenvalues and frequencies between corresponding vibrational modes of QTY-variants and native transporters (Figure S9, S10, S11). This suggests that despite the changes in their environment, the fundamental dynamic properties of the transporters are preserved.

**Fig. 3:**
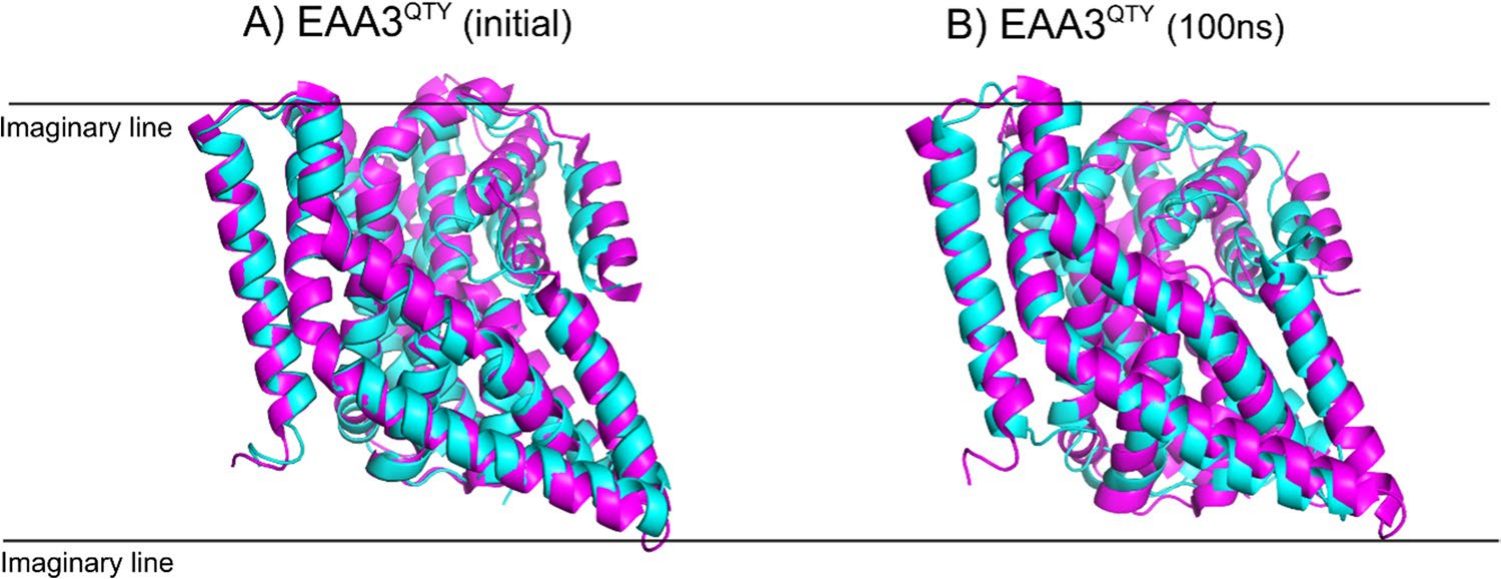
Conformational changes of water-soluble EAA3^QTY^ and experimental outward and intermediate states of native EAA3. Superimposition of EAA3QTY structures from the MD simulation at 100 ns with experimental outward (8CV2) and intermediate (8CV3) states of EAA3. The RMSD values indicate significant structural alignment between the MD-simulated EAA3 and the experimental outward (1.137 Å) and intermediate states (2.347 Å), suggesting consistent conformational behavior. MD simulations were conducted for solutions, with Monte-Carlo placed K^+^ CL^−^ ions (neutralizing, concentration = 0.15 M) (Methods). For clarity, the N- and C-termini and large loops were deleted.

**Fig. 4:**
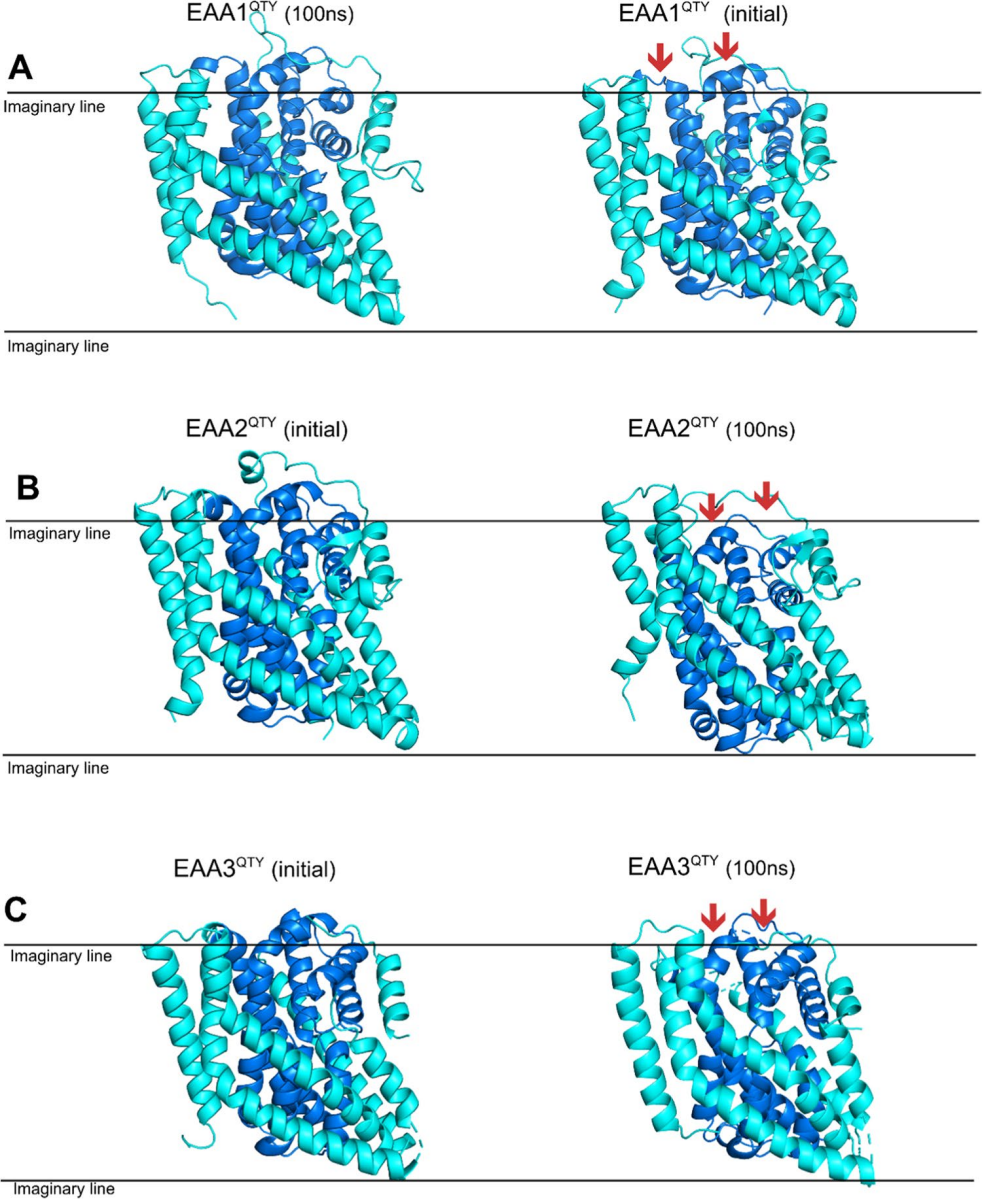
Conformational changes of water-soluble QTY-variants EAA1, EAA2 and EAA3 towards inward states. Conformational changes resembling relatively outward-facing structures (OFS) or inward-facing structures (IFS) from 100ns MD of water-soluble EAA1^QTY^(**A**), EAA2^QTY^(**B**), and EAA3^QTY^(**C**) are visualized. In IFS, the core (darker blue) is located more inside (red arrows) of the peripheral domains of the protein (cyan), relative to the OFS. MD simulations were conducted for solutions, with Monte-Carlo placed K^+^ CL^−^ ions (neutralizing, concentration = 0.15M) (Methods). For clarity, the N- and C-termini and large loops were deleted.

Native transporters and their water-soluble variants exhibit similar intrinsic conformational flexibility, regardless of their environments, as evident from normal modes analysis. The intrinsic similarities and water accessibility, which resemble lipid contacts, may have resulted in the conserved conformational features. The starting structure of EAA1^QTY^ was slightly more inward compared to EAA2 and EAA3. Reflecting this, EAA2-3^QTY^ exhibited a fast IFS trend after 100 ns, contrasting with the outward orientation trend seen in EAA1. This difference is particularly notable in the transmembrane (TM) 1–2 residues, where EAA2^QTY^ and EAA3^QTY^ showed a significant inward trend, with end structures after 100ns MD closely resembling experimental intermediate models of the EAA3 (Fig. 3). In contrast, EAA1^QTY^ did not exhibit a fast movement even after 300 ns of MD simulation, maintaining a configuration more similar to its initial state. As the simulation progressed, EAA1^QTY^ transitioned from a more outward orientation at 100 ns to a more inward configuration by the 220-ns mark, aligning with the behavior of its fellow subfamily members (Fig. 4, Figure S12). This delayed and less profound transition suggests that while EAA1^QTY^ shares the overall conformational flexibility of the other variants, its starting position and possibly inherent structural features lead to a slower transition.

### Comparative Evolutionary Analysis

Glutamate transporters EAA1, EAA2 and EAA3 have retained fundamental structural elements and functional properties through evolutionary scales, despite environmental variations [4, 19]. In addition to the static similarities, our study focuses on the dynamic structural properties of these proteins. These characteristics may act as selection pressures to retain and evolve the functional properties. Subsequently, the residue-wise RMSD values of MD simulations were indeed related to the evolutionary conservation profiles of the residues (Figures S13, S14, S15). Specifically, in the case of the EAA1^QTY^, EAA2^QTY^, EAA3^QTY^ proteins, significant (*p* < 0.001) correlations were observed: rs = 0.37, rs = 0.36, and rs = 0.385, respectively (Figure S16).

**Fig. 5:**
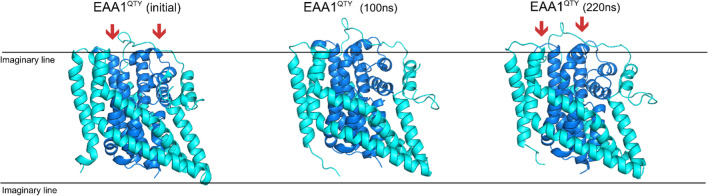
Conformational changes of water-soluble EAA1^QTY^ through 220ns MD (B). Outward-facing structures (OFS) and inward-facing structures (IFS, core is located more inside of the protein relative to the OFS) of water-soluble EAA1^QTY^. Initial structures and structures obtained after 300ns MD simulation are visualized. The MD simulations were conducted for solutions, with Monte-Carlo placed K^+^ CL^−^ ions (neutralizing, concentration = 0.15M) (Methods). For clarity, the N- and C-termini and large loops were deleted.

Native transporters and their water-soluble variants exhibit similar conformational flexibility, enabling them to respond to changes in their surroundings and perform their biological functions effectively. However, it's important to note that factors such as clustering around native sequences at multiple sequence alignment (MSA) generation can complicate evolutionary interpretations [19, 20]. Our previous studies revealed a distinct co-evolution pattern of the QTY-code pairs containing analogous functional regions, such as the T/V substitutions in amine transporters (r = 0.3721, *p* < 0.001) [20]. Hence, whether the conformational flexibility seen in this study resulted from the explained selection pressures or the confounding factor of the co-evolution is hard to distinguish. Further analysis, including experimental evolution studies, could provide valuable insights. Regardless of the underlying evolutionary relationships, our molecular dynamics (MD) simulations revealed that QTY-variants of glutamate transporters can undergo conformational changes similar to native transporters, transitioning between outward and intermediate structures (Fig. 5).

### Future Scopes and the Potential Applications

Despite differences in their environments, native transporters and their water-soluble variants exhibited similar dynamic behaviors, characterized by the analysis of MD simulations, NMA analysis and solvent accessibility profiles. Our primary focus was not on achieving high absolute correlation values but on understanding the initial comparative dynamics and stability of the native *versus* QTY-variant structures. The observed similarities in the initial structural dynamics between native transporters in lipid bilayers and their water-soluble QTY-variants may suggest a nuanced perspective regarding the role of lipids in protein stability. While lipids undoubtedly play crucial roles in membrane protein organization and stability by providing a hydrophobic environment for protein embedding [2, 26], the findings from this comparative analysis hint at the roles of protein structure and function independent of lipid interactions. Instead, it suggests that other factors such as protein-solvent interactions and intrinsic protein flexibility may also significantly contribute to conformational dynamics. The decision to conduct simulations in the simplified environment was a deliberate choice aimed at isolating the intrinsic properties of the QTY variants from the confounding effects of additional molecular interactions. Meanwhile, future studies should consider more complex environmental conditions to better mimic the physiological context. This would enable a more in-depth assessment of the QTY variants' behavior *in vivo*, thereby enhancing the relevance and applicability of our findings. Additionally, our 100 ns and 300 ns molecular dynamics (MD) simulations provided initial insights into the stability of these structures, but enhanced simulation techniques could be employed to capture larger conformational changes, offering a more comprehensive understanding of these variants.

While AF2 has consistently shown high accuracy in predicting protein structures, particularly with QTY-variants [16–20], the lack of experimental structures for the QTY variants introduces a degree of uncertainty in our structural interpretations. Our strategic focus on mutations within transmembrane regions was based on prior evidence that such substitutions can preserve the structure of the protein while enhancing its solubility in aqueous environments [10–14]. Hence, our study focused on QTY-code amino acids that showed electron density map similarities [10, 11]. However, future studies could benefit from exploring less conserved mutations as negative controls to provide further aspects of designing water-soluble variants. By introducing other polar amino acids that are less likely to maintain structural integrity, we could establish a framework for evaluating the effects of polar substitutions on the native proteins' dynamics.

These findings have several implications for drug design, particularly in relation to inhibitor and antibody design. The robustness of protein structure in QTY-variants, independent of lipid interactions, indicates that drugs can be designed to target these water-soluble variants instead, making drug development more versatile and potentially reducing the complexity. With protein-solvent interactions found to be a contributing factor to the observed conformational dynamics, drug designers can develop molecules that stabilize or modulate these interactions, leading to novel therapeutic strategies. Furthermore, by unraveling the evolutionary correlations of the lipid interactions and protein dynamics, our study may ignite further studies on the significance of lipid bilayers in protein evolution. Although small, increased RMSD values were seen in AlphaFold predicted trimers. Further research could explore how expression patterns and trimerization influence conformational changes in these proteins. Studies on protein–protein interactions of QTY-variants could be beneficial.

Our comprehensive analysis of water-soluble QTY-variants provides insights into the conformational dynamics within the glutamate transporter subfamily. From sequence diversities to MD simulations and evolutionary insights, each analysis indicates a strong conservation of the native structural properties. Meanwhile, relative differences offer insights into how QTY-code mutations influence the structural characteristics of the proteins, particularly in relation to their increased solubility.

In a separate study, the water-soluble CXCR4^QTY^ variant receptor has been used to develop a biomimetic sensing device that has extremely high sensitivity [27]. Without making the water-soluble CXCR4^QTY^ variant receptor and to obtain large quantities, it is impossible to develop such applications.

Recently, we also experimentally demonstrated that a designed bacterial membrane protein histidine kinase using the QTY code, it became water-soluble; the histidine kinase not only retained its intact structure, but it also retained its four biological functions, exhibiting expected biophysical properties and highly preserved native molecular function, including the activities of (i) autokinase, (ii) phosphotransferase, (iii) phosphatase, and (iv) pH and potassium signaling [27]. Since histidine kinases are uniquely bacterial enzymes, the water-soluble variant histidine kinases could be used as a target for discovery of a new class of antibiotics to combat widely spread bacterial resistance in hospitals around the world.

Therefore, it is likely the water-soluble EAA1^QTY^, EAA2^QTY^, EAA3^QTY^ variants may also find various application in the future when additional focused research will be carried out.

## Methods

### Protein Sequence Alignments and other Characteristics

Protein sequences for EAA1, EAA2 and EAA3 were retrieved from UniProt (accession numbers P43003, P43004, P43005) (https://www.uniprot.org) [28]. Membrane topology features were plotted using Protter web application (https://wlab.ethz.ch/protter/) [29]. Molecular weights, amino acid compositions, and isoelectric points of native transporters and QTY-variants were calculated using the Expasy tools (https://web.expasy.org/) [30–32]. For the water soluble QTY-variants, topology features were predicted utilizing the TMHMM web server and compared with transmembrane domain predictions for native transporters [33].

### Comparative Structural Analysis

Previously predicted structures of native and QTY-variant monomers were utilized in this study to achieve consistency. The structure predictions of native and QTY-variant monomers were derived from Karagöl et al. [19]. In these predictions, the AlphaFold2 program [34, 35] (https://github.com/sokrypton/ColabFold) was used for the multimer predictions of the transporters following the instructions on the website. Accordingly, for this study, Alphafold2_multimer_v3 was applied for trimer predictions via the same open-source ColabFold pipeline, default parameters were utilized [35].

The experimentally-determined trimers used in this study are: EAA1 outward (PDB ID: 5LLU) [3], EAA2 inward (PDB ID: 7VR8) [5], and EAA3 outward (PDB ID: 8CV2) [2], which were obtained from the RCSB PDB database (https://www.rcsb.org) [36–38]. The structures were superposed using PyMOL Molecular Graphics System version 2 (https://pymol.org/2/) [39], the similarities with experimental structures benchmarked quantitatively by the calculation of root mean square deviation (RMSD) values. For complex molecules like trimers, the large molecule size may result in disparities in RMSD [40] Hence, default 4-cycle outlier reductions were also included along with the all-atom RMSDs (cycles = 0), ensuring the accuracy of the comparisons. Structural superposition with experimental structures further quantified the conformational similarities of predicted multimers. All 5 models of each AlphaFold multimer prediction were assessed for their conformational states.

### Lipid Interactions and Relative Solvent Accessibility (RSA) Calculations

MemProtMD (https://memprotmd.bioch.ox.ac.uk/) was utilized to obtain pre-simulated membrane protein systems embedded in lipid bilayers [41]. The MemProtMD pipeline uses 1000ns Coarse-Grained MD simulations of a lipid bilayer to self-assemble around the experimental model of the transporter [41]. The final 800 ns simulation is then analyzed and converted to atomistic resolution [41]. Since simulations for EAA2 and 5LLU trimer were not available in the database, experimental structures of 8CV2 (EAA3) [2] and 7NPW (EAA1) [42] were used. The pre-simulated data were analyzed for acyl chain dynamics, water contacts, and lipid head contacts within 6Å of the protein over the final 800ns of simulation time. Lipid distortions maps were also obtained, showing the average surface formed by lipid phosphate beads over the 800ns of simulation time. The measurements between lipid heads were obtained from the residue-wise analysis of the MemProtMD database [41]. The annular thickness was visualized, and lipid head distances between layers were measured utilizing PyMOL version 2 [39].

The solvent-accessible surface area (SASA) of the water-soluble QTY-variants was analyzed using PyMOL to assess their exposure to the solvent environment [39]. Residue-wise SASA calculations and secondary structure deductions were analyzed with Stride (https://webclu.bio.wzw.tum.de/cgi-bin/stride/stridecgi.py) [43]. The RSA values of residues were measured using PyMOL by considering their two neighbors, allowing for a more sensitive analysis. To investigate the relationship between the solvent accessibility of EAA1^QTY^ residues and the interactions with lipid molecules, a correlation analysis was performed. The number of lipid head contacts, solvent contacts, and acyl contacts of native EAA1 residues were quantified and correlated with the RSA values of the corresponding EAA1^QTY^ residues. The non-parametric Spearman's rank correlation coefficient (rs) was calculated to assess the strength and direction of the correlations.

### Molecular Dynamics Simulations

The molecular dynamics simulation study was conducted for the AlphaFold2 predicted native transporters and their QTY-variants. The simulations were carried out on Google Compute Engine, using an Ubuntu-based Virtual Machine setup with a total of 172-core Intel Sapphire Rapids CPUs. Configuration files and Linux bash scripts for the simulations have been made publicly available, accompanied by step-by-step instructions. The simulation input files and outputs were stored in the distributed file system provided by Google Compute Engine.

The membrane-protein systems were built for native EAA1, EAA2 and EAA3 using the membrane builder of the CHARMM-GUI web server [44–47], with AlphaFold2 predicted structures. The protein component was placed at the center of a rectangular box. The PPM 2.0 method, which considers the anisotropic water–lipid environment, was used to optimize the spatial positioning of the proteins relative to the lipid bilayer [48]. The membrane was composed of 70% 1-palmitoyl-2-oleoyl-glycero-3-phosphocholine (POPC) and 30% cholesterol. The system was solvated in TIP3P water with 150mM KCl. On the other hand, the primary focus of this study is the dynamics of QTY-variants. As prior topology analysis indicated, these variants are unable to locate in the lipid bilayer, solution-protein systems were built instead, using the solution builder of the CHARMM-GUI web server [45–47], with AlphaFold2 predicted structures. System pH was set to 7.0. The rectangle-shaped water box of edge distance 10Å was formed and solvated with water using explicit solvation. The system was built with K^+^, Cl^−^ ions in a concentration 0.15M (neutralizing). The initial configuration of ions was determined through Monte Carlo (MC) simulations (2000 steps) using a primitive model.

All molecular dynamics (MD) simulations were conducted with GROMACS 2022.3 [49], utilizing the all-atom CHARMM36m [50] force field. The system's energy was minimized using the steepest descent method until the maximum forces were below 1000 kJ/mol/nm. Electrostatic interactions were handled with Particle Mesh Ewald (PME), and a cutoff of 1.2 nm was applied for both Coulombic and van der Waals interactions. A multi-step minimization and equilibration procedure was employed to relax the protein-membrane systems and achieve stable equilibrium. Equilibration was carried out for 125 ps using the standard CHARMM-GUI protocol [45, 47]. The Parrinello-Rahman barostat with semi-isotropic coupling and the Nose–Hoover thermostat was used. The temperature was held at 303.15 K and pressure was held at 1 bar. After NVT and NPT equilibration, 100ns productions and a 300ns production MD simulation was run with timestamps for each 10ns. The trajectories were later combined with the gmx traj tool. The root mean square deviation (RMSD) of the backbone atoms of the protein was calculated using UCSF ChimeraX software version 1.7 (https://www.rbvi.ucsf.edu/chimerax/) [51]. The structures and 100ns trajectories were visualized using PyMOL (https://pymol.org/2/) [39]. Since existing experimental models for EAA1 and EAA2 are only available in a single state, the conformational changes of EAA3^QTY^ were compared with the experimental models of different conformational states of EAA3, namely the outward state (PDB ID: 8CV2) [2] and the intermediate state (PDB ID: 8CV3) [2].

### Normal Mode Analysis

Normal mode analysis was performed using the Bio3D package to assess the dynamic properties of native transporters and their QTY-variants [52]. The analysis and visualization were performed on Google Collaboratory with a Jupyter Notebook in R language [53]. NMA was conducted to calculate the eigenvalues and frequencies of the vibrational modes of both native transporters and QTY-variants. The resulting eigenvalues and frequencies were compared between corresponding vibrational modes to evaluate the similarities in their dynamic properties.

### Evolutionary Conservation Profiles

ConSurf server [54–57] (https://consurf.tau.ac.il/) was used for generating evolutionary conservation profiles. The server was run with AlphaFold2 predicted native structures that were also used for RMSD calculations. The source sequences for the native structures were derived from Uniprot, as in our previous study [19]. The conservation scores were also generated according to AlphaFold2 predicted QTY-variant structures. The conservation scores were computed using the Bayesian method, with the amino acid substitution model chosen based on the best fit, and the default parameters on the server were used. The conservation grades and residue exposure data obtained from the ConSurf server were correlated with the residue-wise RMSD values between the initial structures and the final 100ns MD simulation, which was visualized using the UCSF ChimeraX software version 1.7 [51].

### Statistical Calculations

The Shapiro–Wilk normality test was performed and showed that the distribution of the scores departed significantly from normality [58]. Because of the violation of the assumptions of linearity and normality for the variables, nonparametric analyses were performed, and bootstrapping was used in the analyses to calculate confidence intervals [59, 60]. The Spearman's rank correlation coefficient (Spearman's ρ) was utilized to measure the monotonic association between two variables, providing a more robust approach in the presence of non-linear relationships [60, 61]. Moreover, the variables might have a non-linear relationship and contain outliers, which Pearson’s coefficients are more sensitive of [61].

A Multivariate analysis was utilized to remove the effects of different confounding variables and to isolate the specific impact of QTY modifications on protein-lipid interactions. Water soluble residues of native transporters were retained in QTY variants, and the substitutions occurred in hydrophobic residues. This may result in a confounding effect towards water contacts. Since homoscedasticity assumptions may not be met by our non-parametric data, the Spearman partial rank-order correlation coefficients were used as the multivariate analysis [61, 62]. Statistical calculations were performed and visualized using R (The R Foundation for Statistical Computing, Vienna, Austria), version 4.3.1 (https://www.r-project.org/) [53].

## Supplementary Information

Supplementary data file containing detailed analyses on AlphaFold predictions, lipid contact maps and benchmarking, MD analysis and statistical analyses, and comparative graphs supporting the main findings presented in the manuscript.

Supplementary file1 (PDF 11.1 MB)

## Data Availability

Each statistical and computational analysis of this study, included with step-by-step instructions where possible, are publicly available to ensure repeatability. For more detailed information on the statistical analyses, input files and detailed outputs, including the AlphaFold2 calculations and codes to regenerate analyses, please visit the website: https://github.com/karagol-alper/QTY-dynamics-EAA13. Further information and requests for data should be directed to and will be fulfilled by A.K. alper.karagol@gmail.com, and T.K. taner.karagol@gmail.com.
