# Supplementary material for: Molecular Dynamic Simulations Reveal that Water-Soluble QTY-Variants of Glutamate Transporters EAA1, EAA2 and EAA3 Retain the Conformational Characteristics of Native Transporters
Source: Pharm Res. 2024 Sep 25;41(10):1965–77. doi: 10.1007/s11095-024-03769-0 (PMC11530497; doi:10.1007/s11095-024-03769-0)
Supplement: Supplementary file 1 — Supplementary file1 (PDF 11.1 MB) [file 11095_2024_3769_MOESM1_ESM.pdf]

Supplementary Information

Molecular Dynamic Simulations Reveal that Water-Soluble QTY-Variants of Glutamate Transporters EAA1, EAA2 and EAA3 Retain the Conformational Characteristics of Native Transporters

Alper Karagöl[1,¶], Taner Karagöl[1,¶], Shuguang Zhang[2,*]

[1]Istanbul University Istanbul Medical Faculty, Istanbul, Turkey

[2]Laboratory of Molecular Architecture, Media Lab, Massachusetts Institute of Technology, 77 Massachusetts Avenue, Cambridge, MA, 02139, USA

[¶]These authors contribute equally.

*To whom the correspondence should be addressed.

Email:
Taner Karagöl, taner.karagol@gmail.com ORCID: 0009-0005-1011-7661
Alper Karagöl, alper.karagol@gmail.com ORCID: 0009-0001-7864-0732
Shuguang Zhang, Shuguang@MIT.EDU ORCID: 0000-0002-3856-3752

Table S1. Characteristics of native transporters of EAA1-3 and their water-soluble QTY variants.

Name	RMSD	pI	MW (KD)	TM variation (%)	Overall variation (%)
EAA1	-	8.52	59.6		
EAA1^{QTY}	0.717Å	8.44	59.9	53.88	17.89
EAA2	-	6.09	62.1		
EAA2^{QTY}	0.948Å	6.09	62.4	48.92	15.85
EAA3	-	5.56	57.1		
EAA3^{QTY}	0.905Å	5.56	57.4	50.27	17.74

Table derived from Karagöl et. al (2024A). Residue mean-square distance (RMSD) in Å, Isoelectric focusing (pI), Molecular weight (MW), Transmembrane (TM), - = not applicable. The internal and external loops have no changes, the overall changes are significant, and the TM changes are rather large. (Methods).

Table S2. RMSD between AlphaFold2 predicted monomers, and cryo-EM/crystal structures.

Name	PDB ID	RMSD$^{Native/Experimental}$	RMSD$^{QTY/Experimental}$
EAA1 (outward)	5LLU (outward)	0.920Å	1.180Å
EAA3 (outward)	8CV2 (outward)	1.141Å	1.339Å

All RMSD values are below 3Å and show good superposition between structures.

Table S3. Conformations of AlphaFold2 Multimer predicted native transporter assemblies, and RMSD between experimental cryo-EM/crystal structures.

Name[1]	Model ID	Relative Conformation of the Model[2]	RMSD (excluding outliers)[3]	All-atom RMSD
**EAA1 (5LLU)**	Homotrimer_modelrank002	Outward	1.703Å (69)	3.11Å
	Homotrimer _modelrank001	Outward	1.946Å (43)	5.88Å
	Homotrimer _modelrank005	Inward	9.336Å (18)	9.91Å
	Homotrimer _modelrank004	Inward	9.552Å (24)	10.23Å
	Homotrimer _modelrank003	Outward	33.117Å (0)	33.117Å
**EAA2 (7VR8)**	Homotrimer _modelrank005	Inward	1.499Å (243)	2.82Å
	Homotrimer _modelrank004	Inward	1.554Å (190)	3.98Å
	Homotrimer _modelrank003	Outward	33.671Å (0)	33.671Å
	Homotrimer _modelrank001	Outward	33.790Å (3)	33.99Å
	Homotrimer _modelrank002	Outward	9.898Å (38)	10.58Å
**EAA3 (8CV2)**	Homotrimer _modelrank001	Outward	1.208Å (41)	1.67Å
	Homotrimer _modelrank003	Outward	1.332Å (21)	2.06Å
	Homotrimer _modelrank002	Outward	1.540Å (15)	2.16Å
	Homotrimer _modelrank005	Inward	34.302Å (3)	34.47Å
	Homotrimer _modelrank004	Outward	35.403Å (0)	35.403Å

[1]Model that compared with AlphaFold multimer predictions, each model is experimentally derived trimer assemblies of the transporter. PDB IDs of experimental structures is in parenthesis.
[2]Relative conformation of the model according to protein core located more inward or outward.
[3]RMSD after 4 -cycle outlier reduction, outlier atoms counts are in parenthesis (Methods).

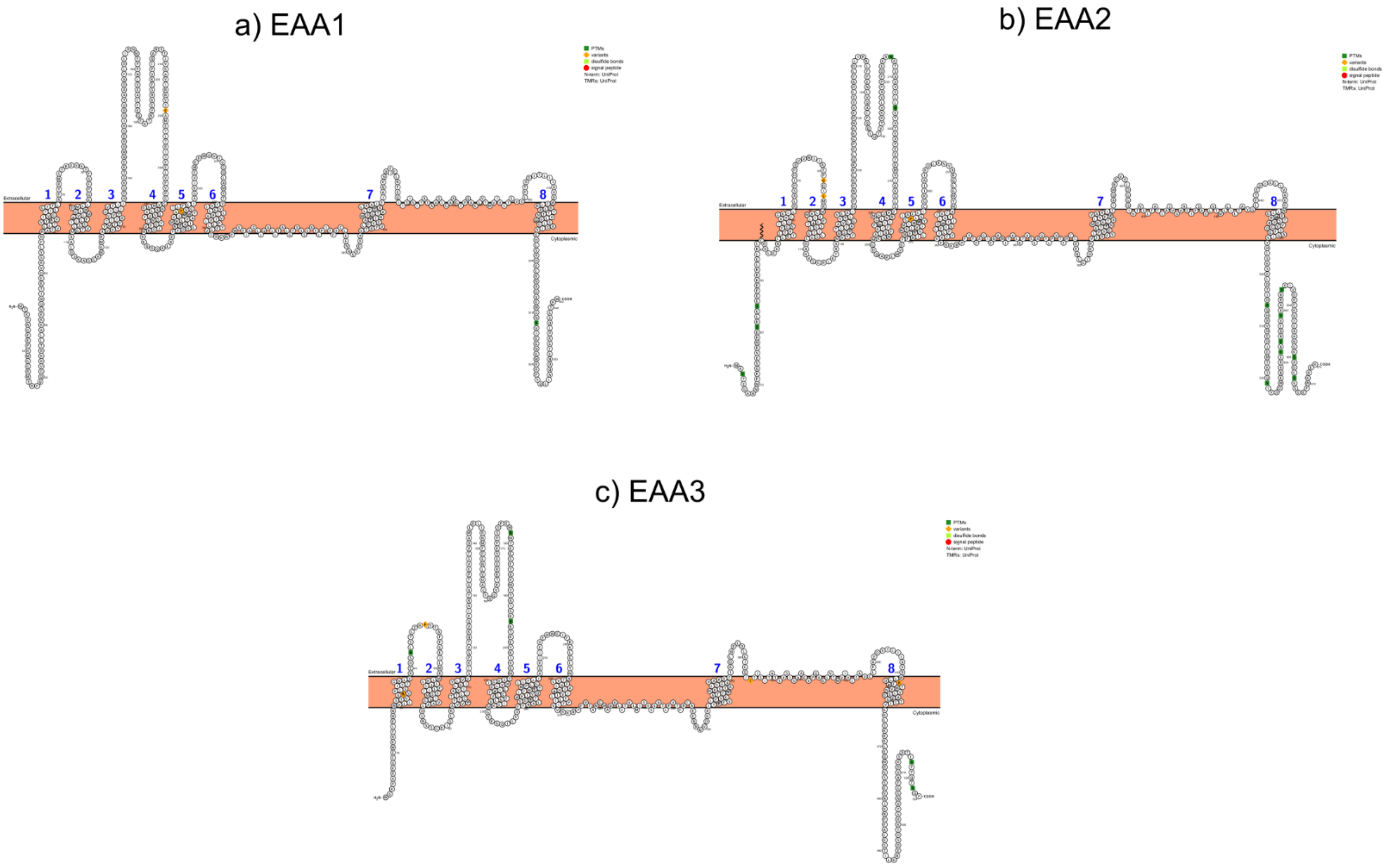

Figure S1. Membrane topology models of EAAT1-3. Prediction was performed with the Protter web client, based on the sequence analysis of the corresponding transporter using the UniProt database. Each transporter has an 8-transmembrane (TM) architecture.

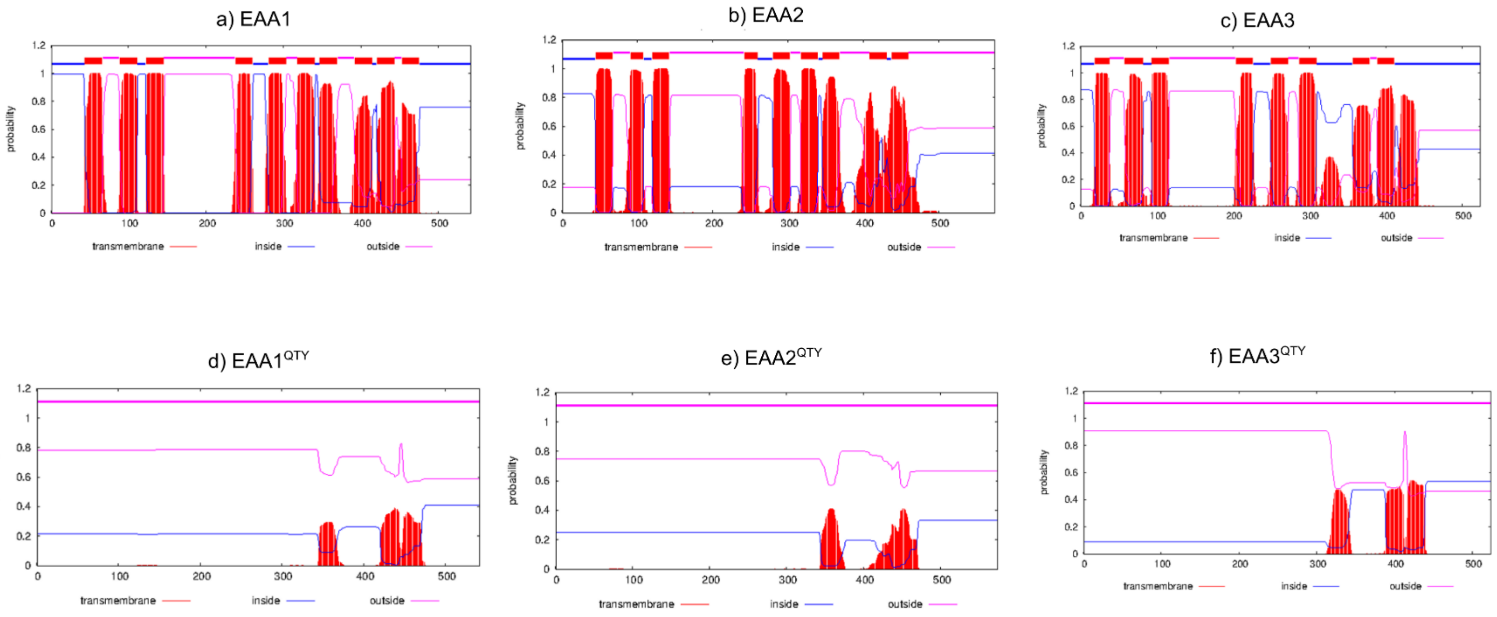

Figure S2. Transmembrane helices (TMH) predictions of water-soluble QTY variants EAA1-3. Predictions were performed with the TMHMM web client. Each transporter has an 8-transmembrane (TM) architecture (a, b, c). The number of predicted TMH for QTY variants were zero (d, e, f).

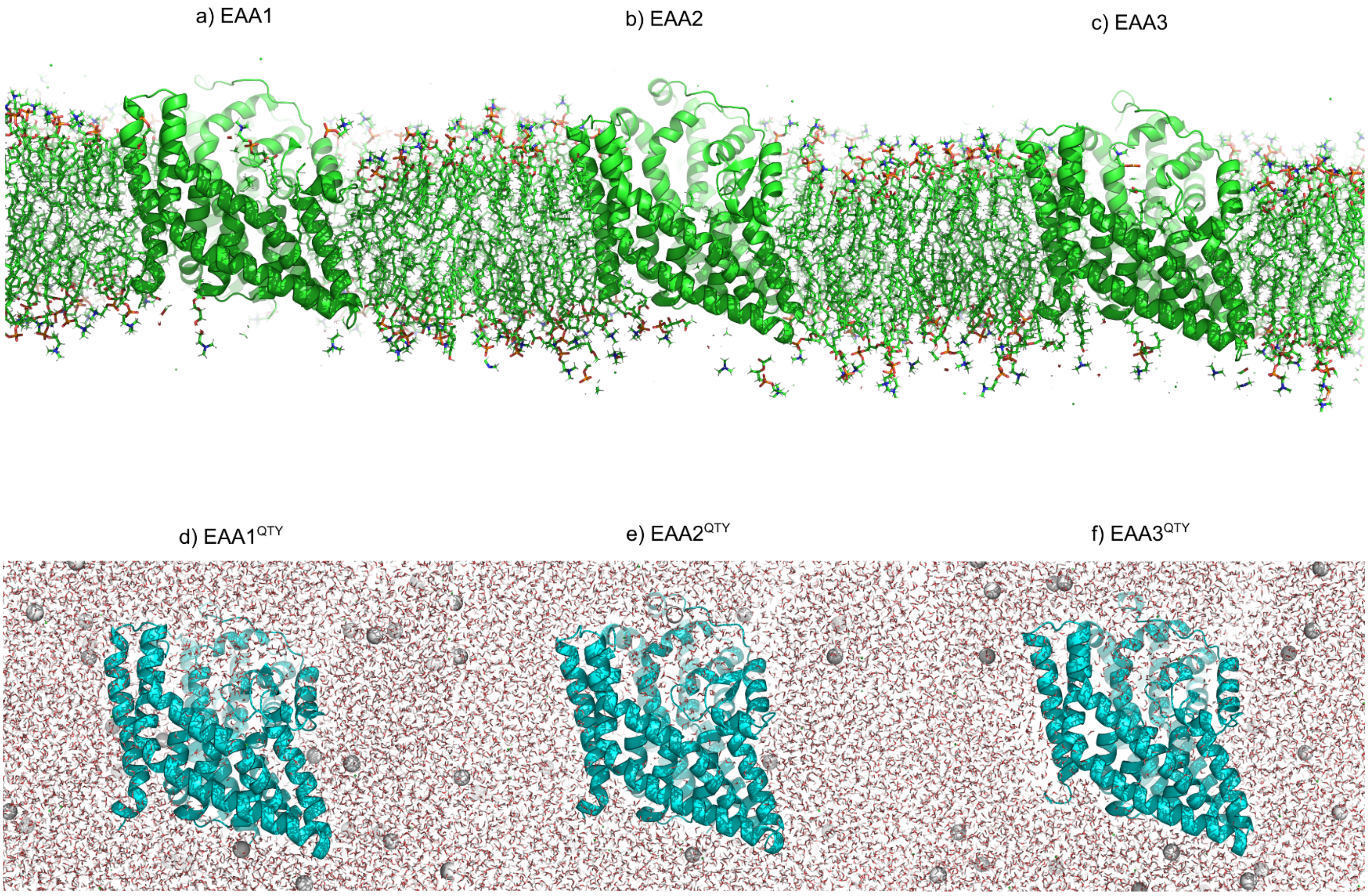

Figure S3. AlphaFold2 predicted EAAT1-3 monomers in lipid bilayer and their QTY-variants in water solvent. The complexes of EAA1-3 and their QTY variants after equilibrium MD. For clarity, the N- and C-termini and large loops, which are not resolved in experimental structures, were deleted.

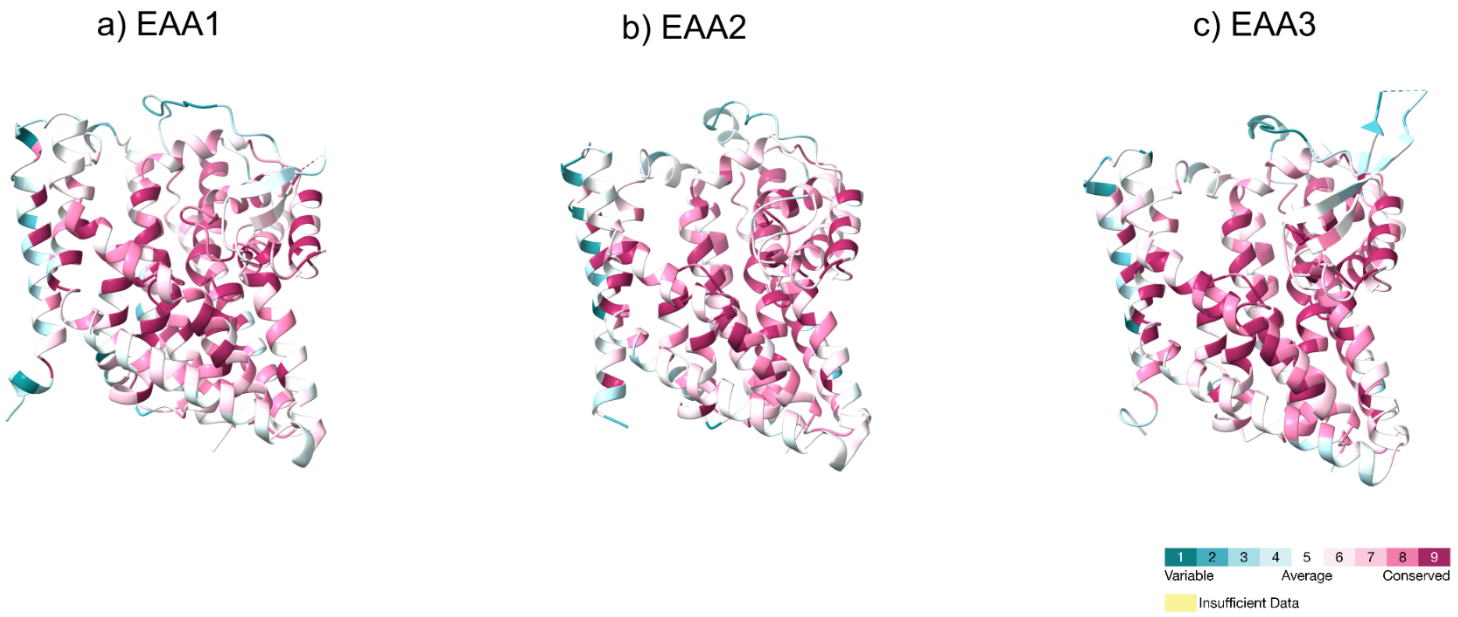

Figure S4. Evolutionary conservation profiles of native EAA1-3. Evolutionary conservation grades of each amino acid residue predicted by ConSurf server; visualized by the color-coding scheme of nine colors, ranging from turquoise (variable) through white (average) through burgundy (conserved) represents conservation grades 1 to 9, in order of increasing conservation (1= Variable, 5= Average, 9= Conserved). Conservation grades were calculated for the source amino acid sequence and the corresponding Alphafold2 predicted native structure.

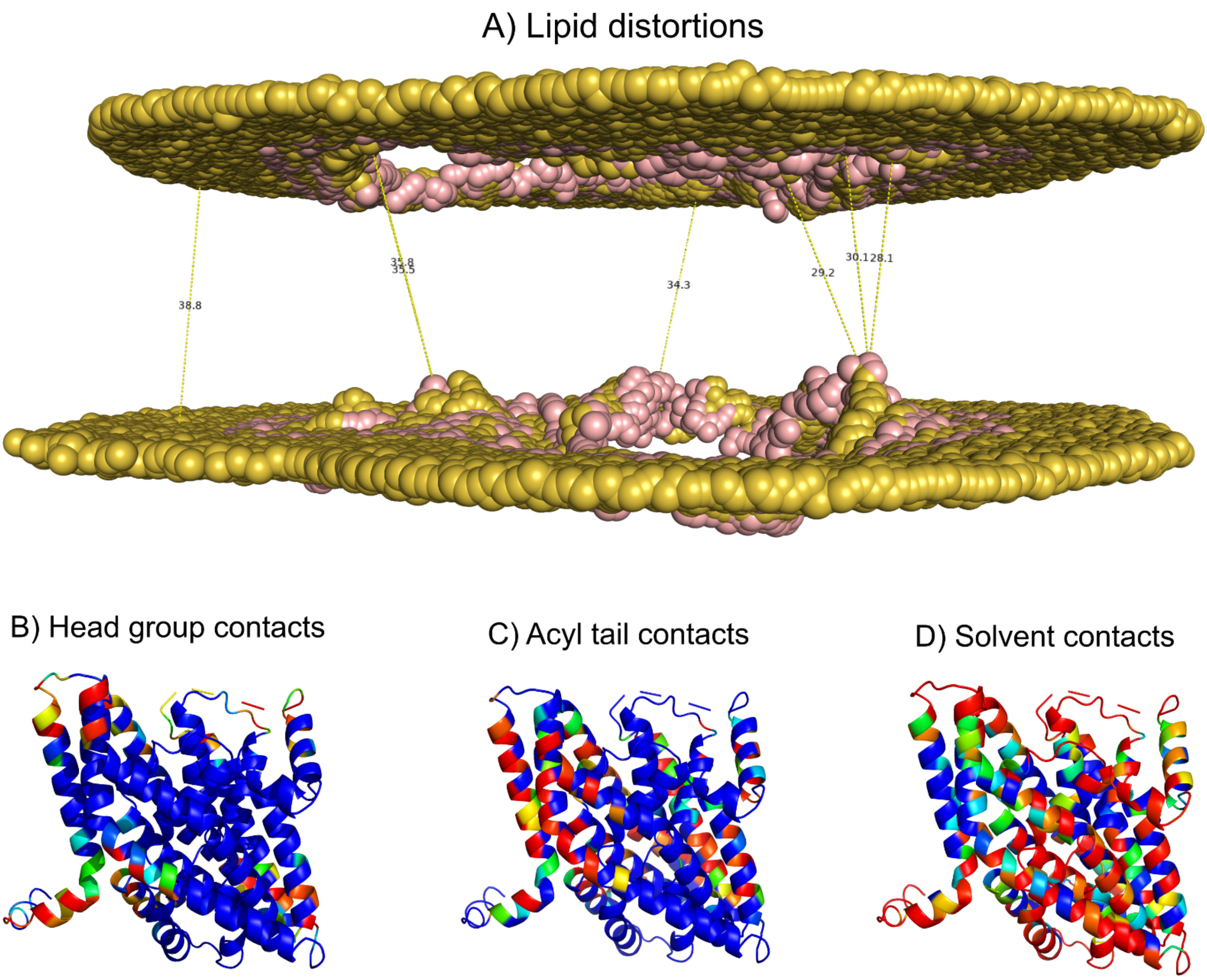

Figure S5. Lipid Distortions of experimental EAA1 bio assembly (7NPW) structure through 800ns MD simulation. At the point of maximum closure, the lipid heads of both the upper and lower leaflets approached within a distance as close as 28.1Å, deviating from the 38.8Å annular thickness (A). Contacts show the average occupancy of the group within 6 Å of the protein over the final 800ns of simulation time (B, C, D). The contacted residues are colored red and residues that have lesser contacts of the groups are colored blue.

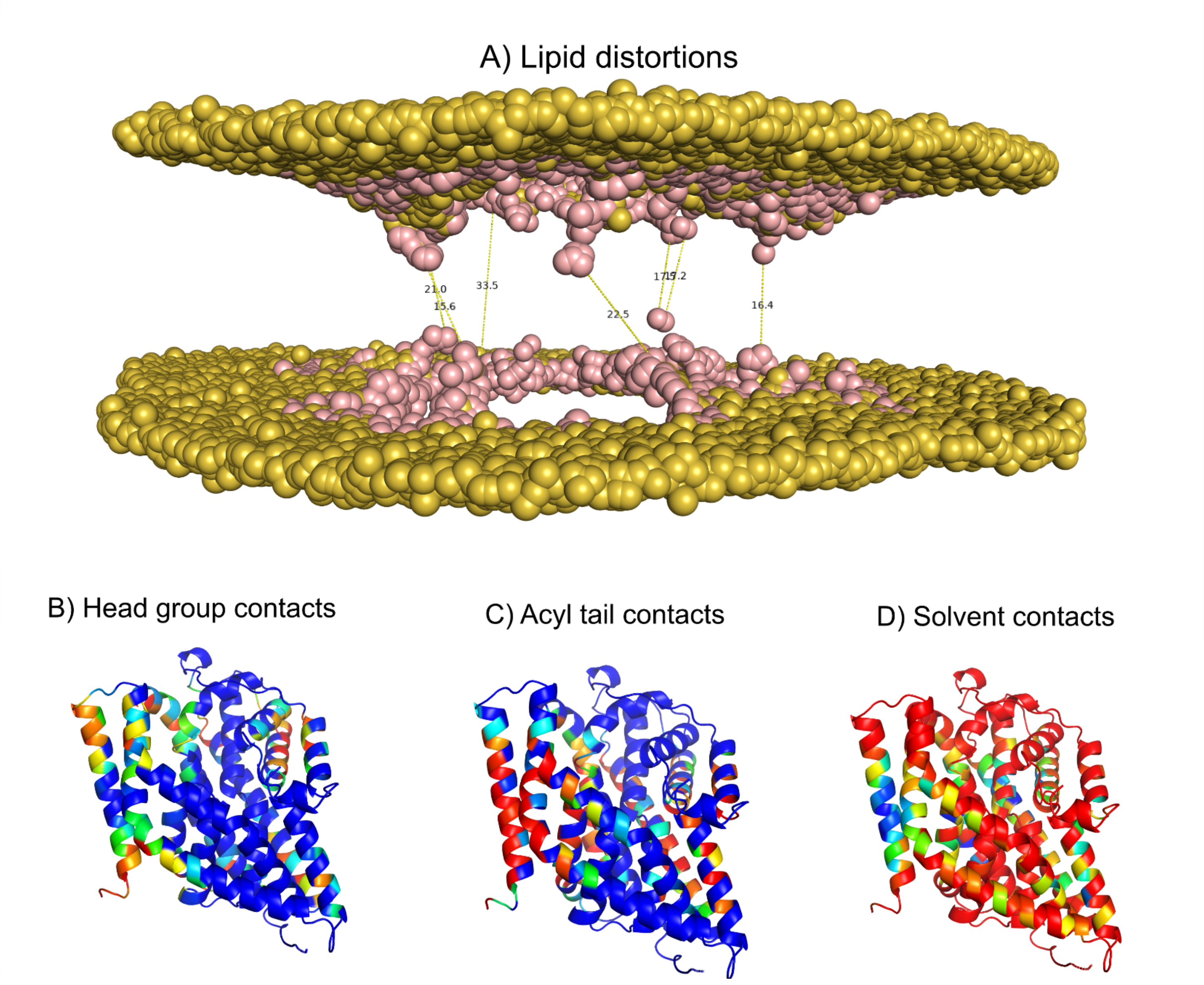

Figure S6. Lipid Distortions of the experimental EAA3 bio assembly (8CV2) structure through 800ns MD simulation. At the point of maximum closure, the lipid heads of both the upper and lower leaflets approached within a distance of 15.6Å, from the 33.5Å annular thickness (A). Contacts show the average occupancy of the group within 6 Å of the protein over the final 800ns of simulation time (B, C, D). The contacted residues are colored red and residues that have lesser contacts of the groups are colored blue.

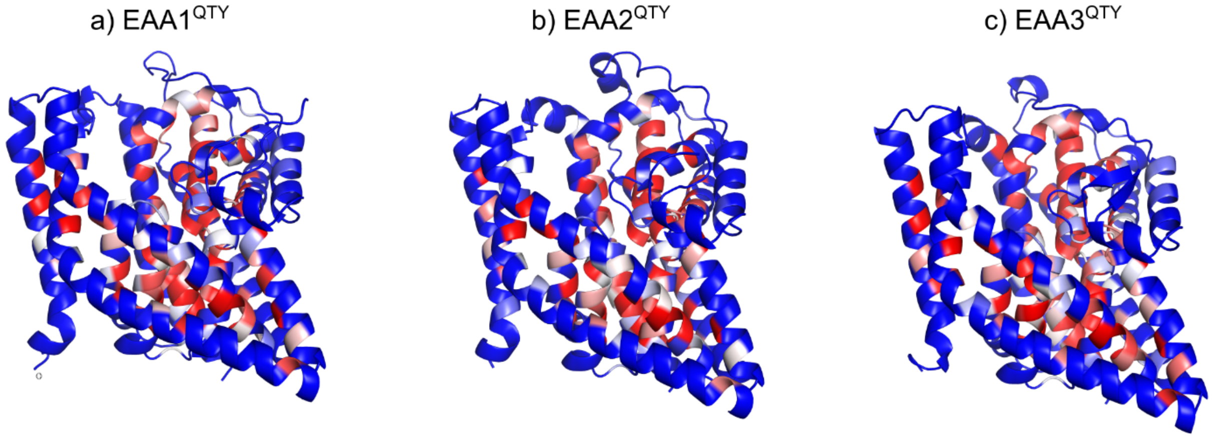

Figure S7. Relative solvent accessibility (RSA) of QTY-variants of EAA1-3. Relative solvent accessibility (RSA) calculated by removing other residues except its two next neighbors. The residues of outer exposed helices of the proteins are relatively more accessible (colored blue) than certain resides of the core helices (red or white).

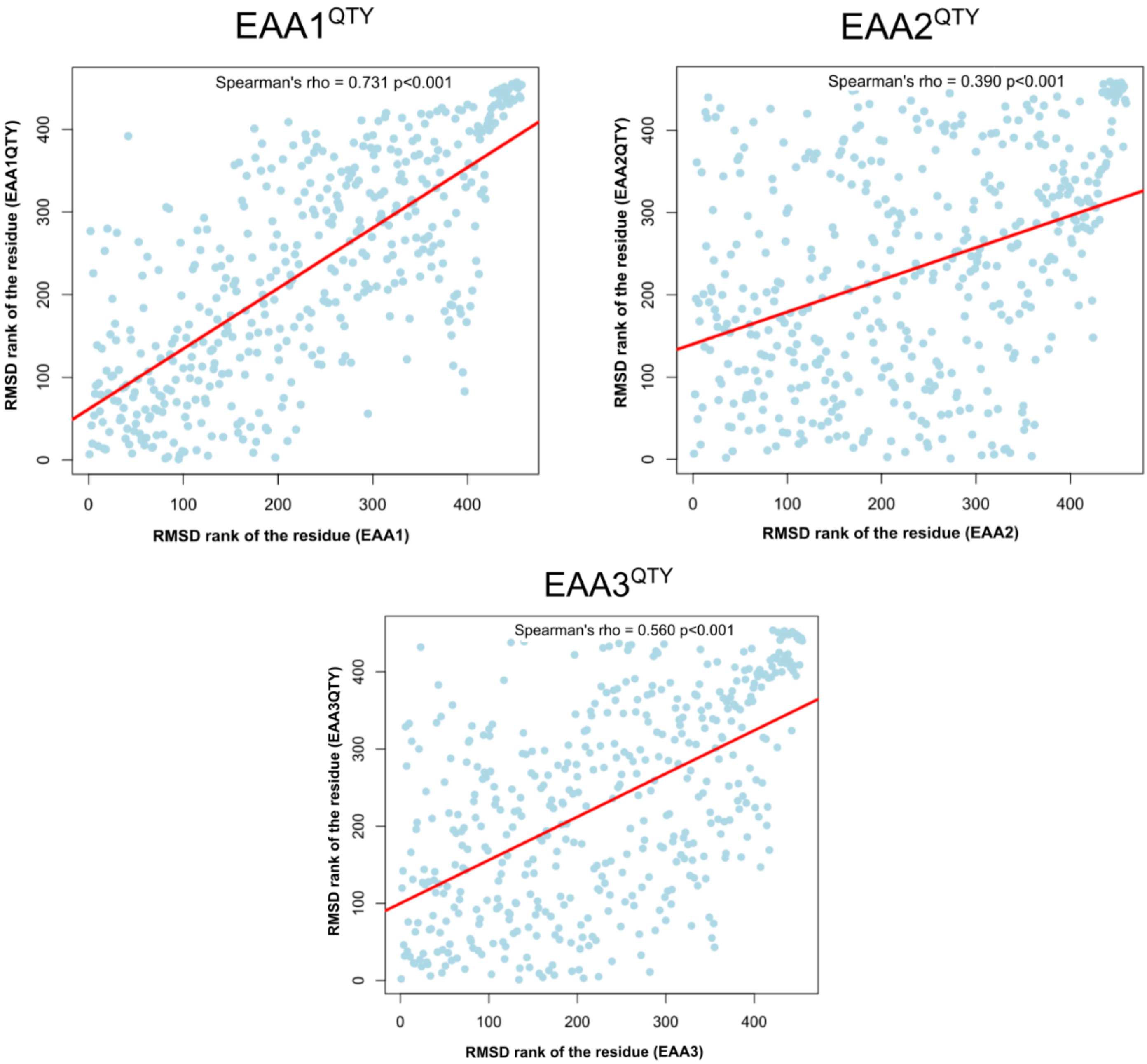

Figure S8. Comparison of residue-wise RMSD fluctuations of native transporters in lipid bilayer and their water-soluble QTY variants. Scatter plot of residue-wise RMSD of the initial structure and structures after conducting 100ns MD. Regression lines are colored red.

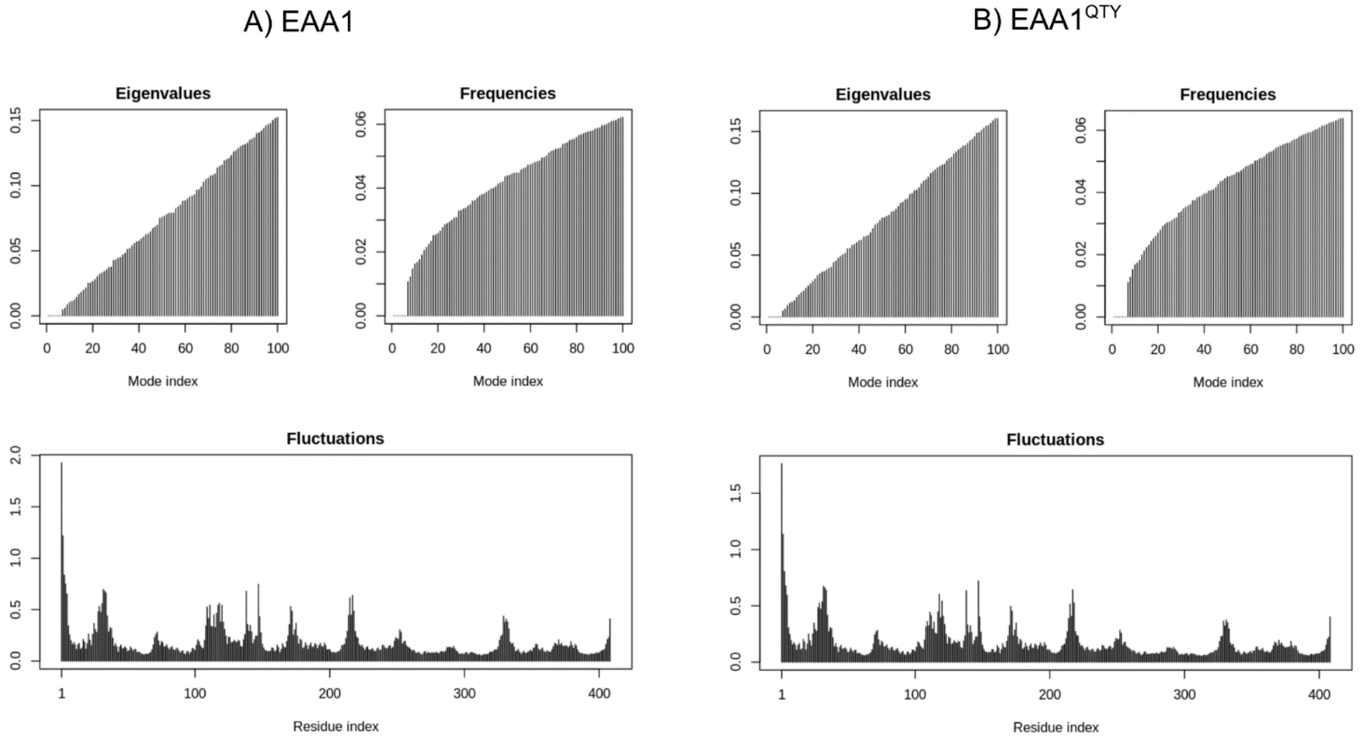

Figure S9. **Vibrational modes of EAA1 and EAA1^{QTY}.** The normal modes analysis (NMA) represents of the eigenvalues and vibrational frequencies of mechanistic modes. The normal modes and their frequencies of EAA1(A) and EAA1^{QTY}(B) are similar.

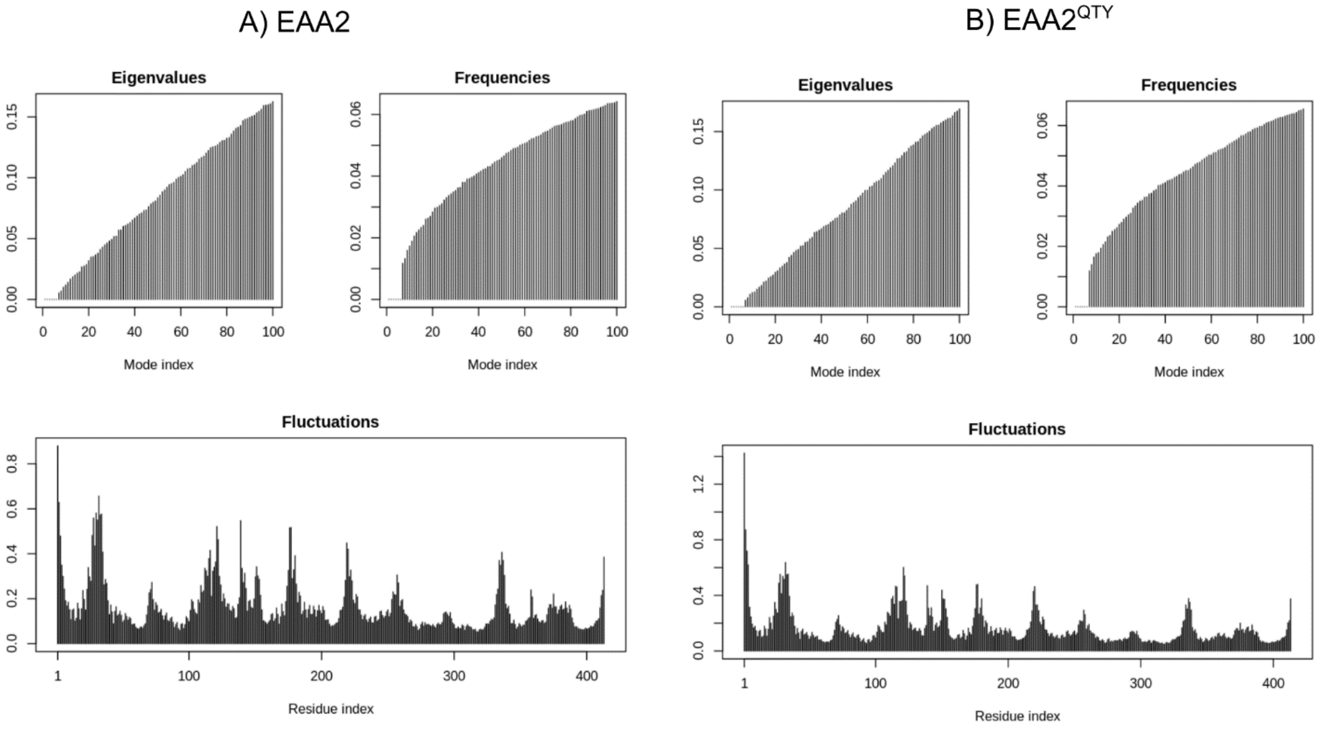

Figure S10. **Vibrational modes of EAA2 and EAA2^{QTY}.** The normal modes analysis (NMA) represents of the eigenvalues and vibrational frequencies of mechanistic modes. The normal modes and their frequencies of EAA2(A) and EAA2^{QTY}(B) are similar.

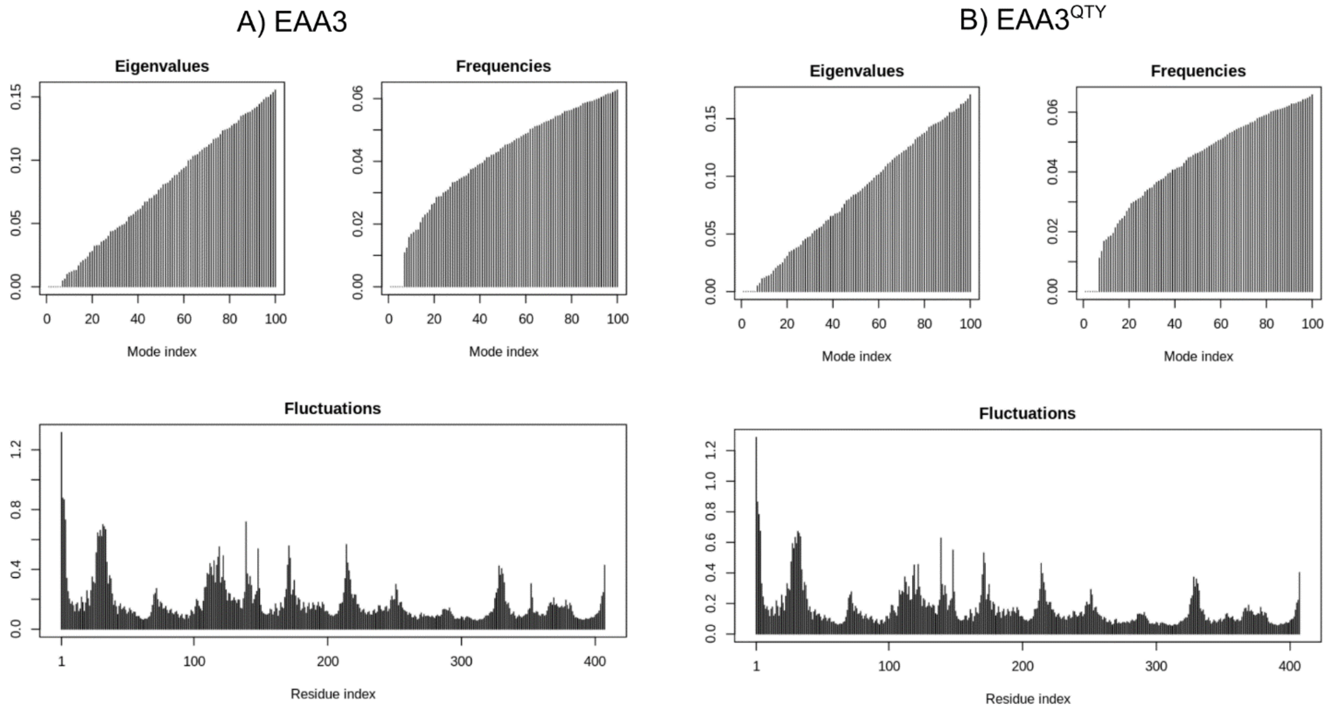

Figure S11. **Vibrational modes of EAA3 and EAA3^{QTY}.** The normal modes analysis (NMA) represents of the eigenvalues and vibrational frequencies of mechanistic modes. The normal modes and their frequencies of EAA3(A) and EAA3^{QTY}(B) are similar.

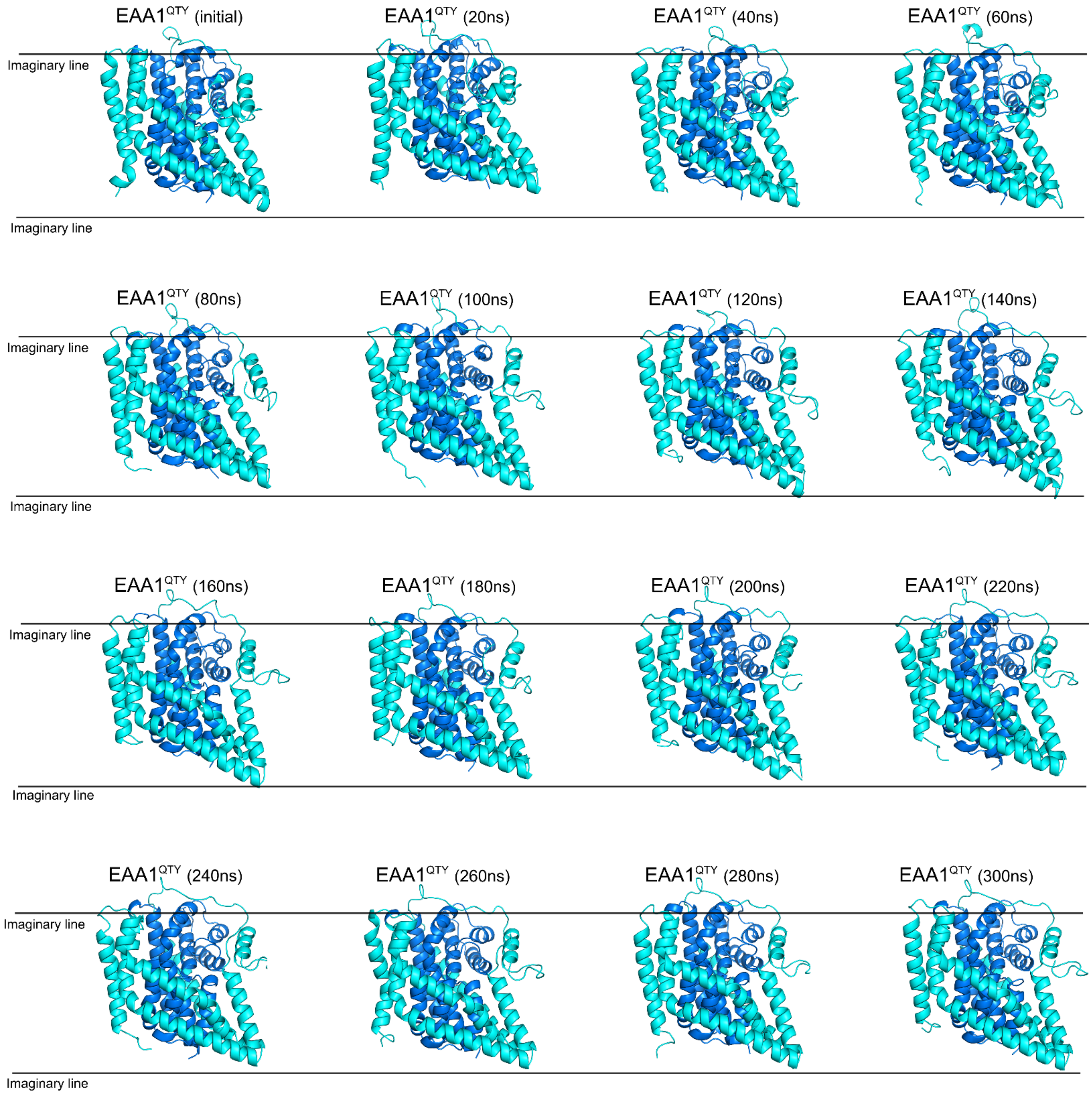

Figure S12. **EAA1^{QTY} structural fluctuation over 300ns MD.** The MD simulations were conducted for solution, with Monte-Carlo placed K^+ CL^- ions (neutralizing, concentration=0.15) (Methods). For clarity, the N- and C-termini and large loops were deleted.

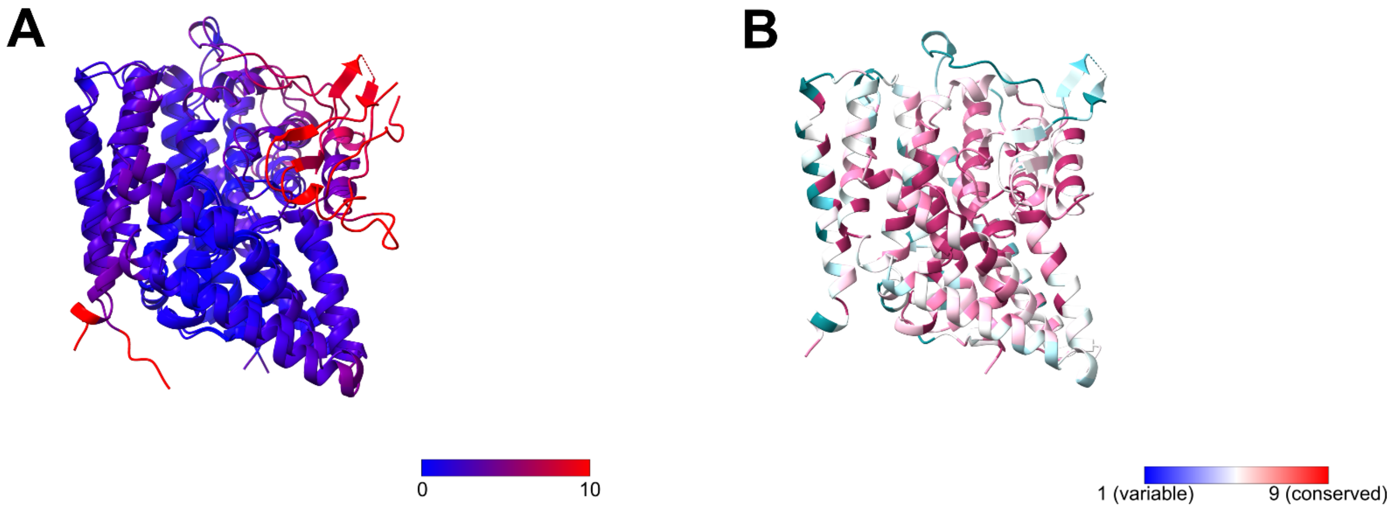

Figure S13. **EAA1^{QTY} residue-wise RMSD fluctuation over 100ns MD (A) and evolutionary conservation profiles (B).** Each residue's RMSD value (0 to 10) indicates the deviation of its position compared to the initial structure over the course of the simulation. Higher RMSD values suggest greater structural fluctuation (for TM helices) or flexibility (for loops). Evolutionary conservation grades of each amino acid residue predicted by ConSurf server; visualized by the color-coding scheme of nine colors, ranging from turquoise (variable) through white (average) through burgundy (conserved) represents conservation grades 1 to 9, in order of increasing conservation (1= Variable, 5= Average, 9= Conserved). For clarity, the N- and C-termini and large loops were deleted.

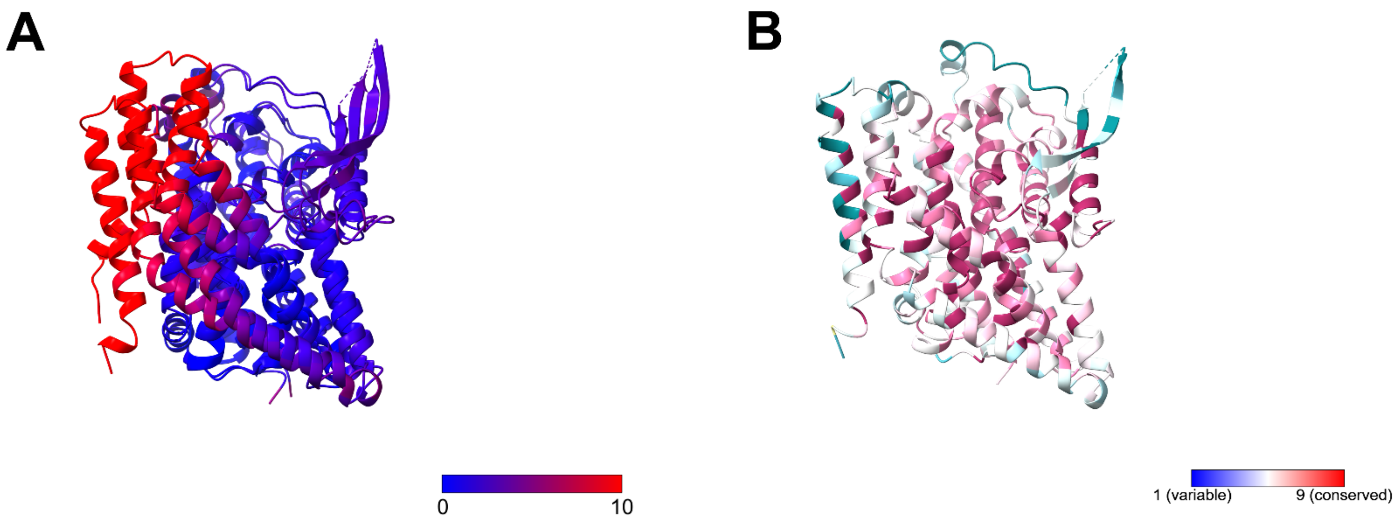

Figure S14. **EAA2^{QTY} residue-wise RMSD fluctuation over 100ns MD (A) and evolutionary conservation profiles (B).** Each residue's RMSD value (0 to 10) indicates the deviation of its position compared to the initial structure over the course of the simulation. Higher RMSD values suggest greater structural fluctuation (for TM helices) or flexibility (for loops). Evolutionary conservation grades of each amino acid residue predicted by ConSurf server; visualized by the color-coding scheme of nine colors, ranging from turquoise (variable) through white (average) through burgundy (conserved) represents conservation grades 1 to 9, in order of increasing conservation (1= Variable, 5= Average, 9= Conserved). For clarity, the N- and C-termini and large loops were deleted.

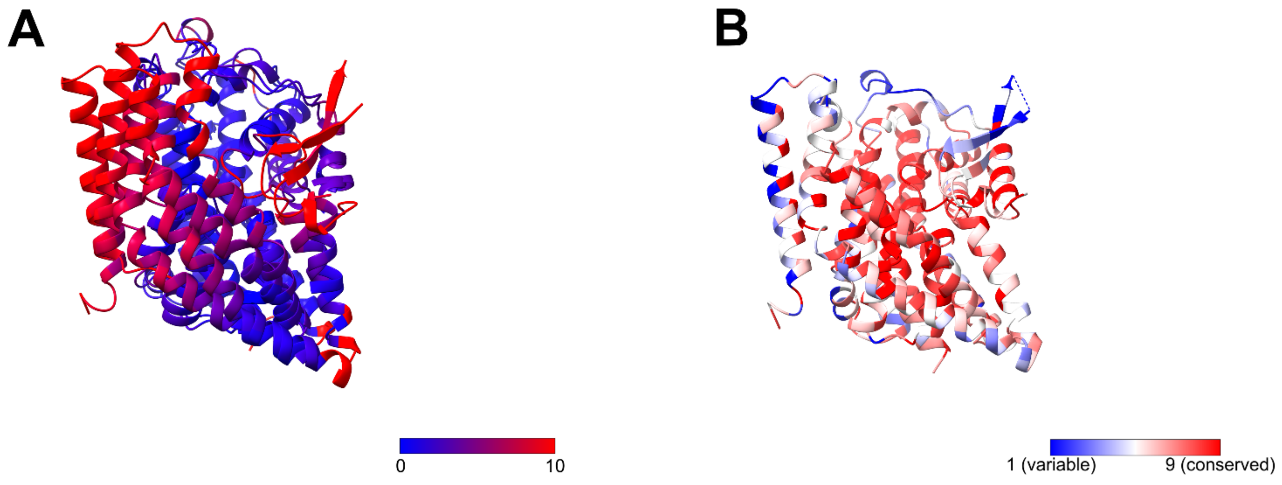

Figure S15. **EAA3^{QTY} residue-wise RMSD fluctuation over 100ns MD (A) and evolutionary conservation profiles (B).** Each residue's RMSD value (0 to 10) indicates the deviation of its position compared to the initial structure over the course of the simulation. Higher RMSD values suggest greater structural fluctuation (for TM helices) or flexibility (for loops). Evolutionary conservation grades of each amino acid residue predicted by ConSurf server; visualized by the color-coding scheme of nine colors, ranging from turquoise (variable) through white (average) through burgundy (conserved) represents conservation grades 1 to 9, in order of increasing conservation (1= Variable, 5= Average, 9= Conserved). For clarity, the N- and C-termini and large loops were deleted.

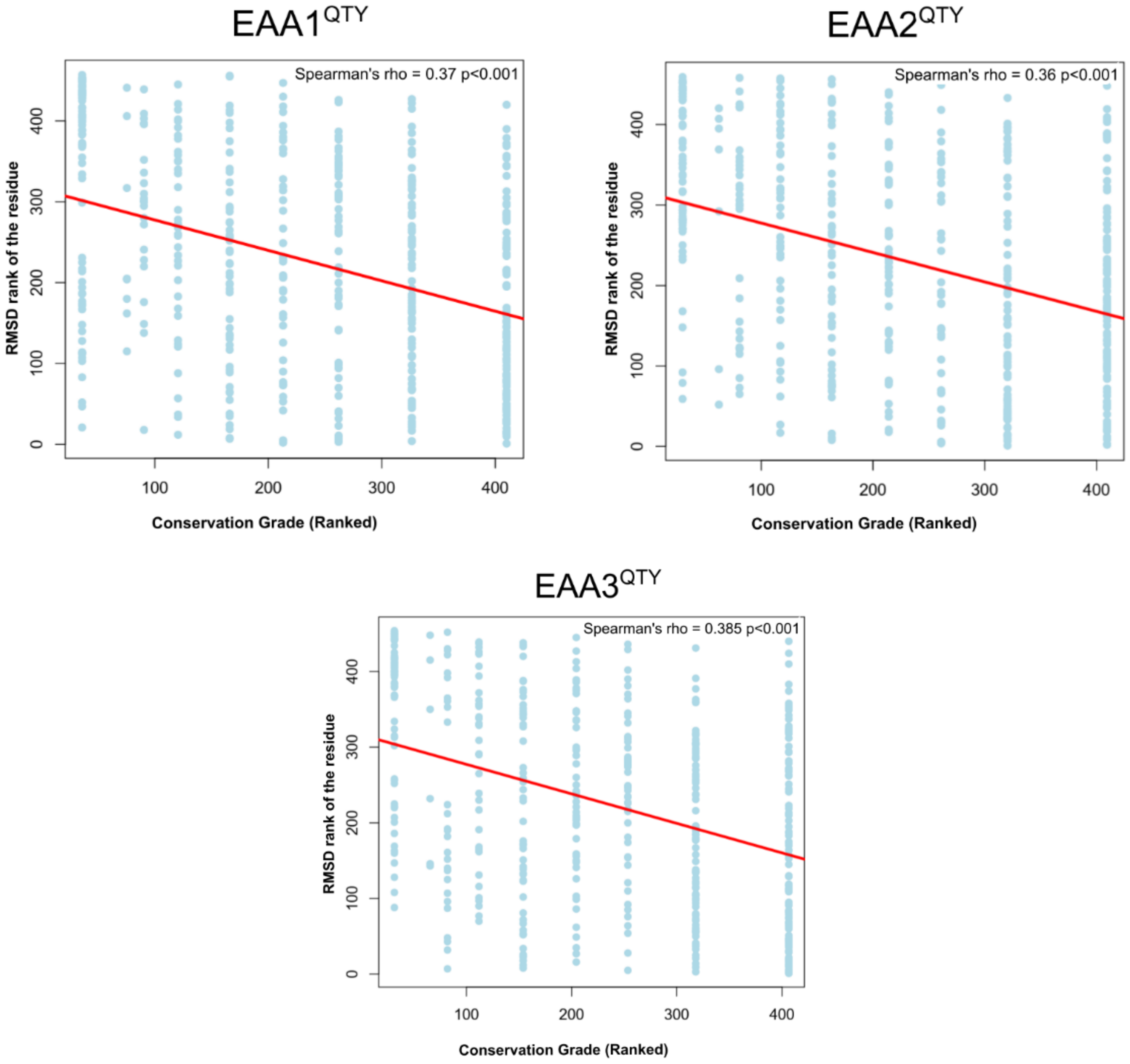

Figure S16. Comparison of residue-wise RMSD fluctuations with evolutionary conservations. Scatter plot of ranked values of ConSurf evolutionary conservation grades and residue-wise RMSD of the 100ns MD. Regression lines are colored red.

Figure S17. Enlarged panel a-c of sequence alignments EAA1-3. The protein sequences of each monoamine transporter are now clearly visible. The QTY variant sequences are below the native protein sequences. The QTY amino acid substitution changes are colored in red. Other colour code: Yellow line-intracellular, Blue wave-transmembrane helices, Pinkish line-extracellular, Green line-peripheral domains and hairpin loops. Figure derived from Karagöl et. al. (2024 A).

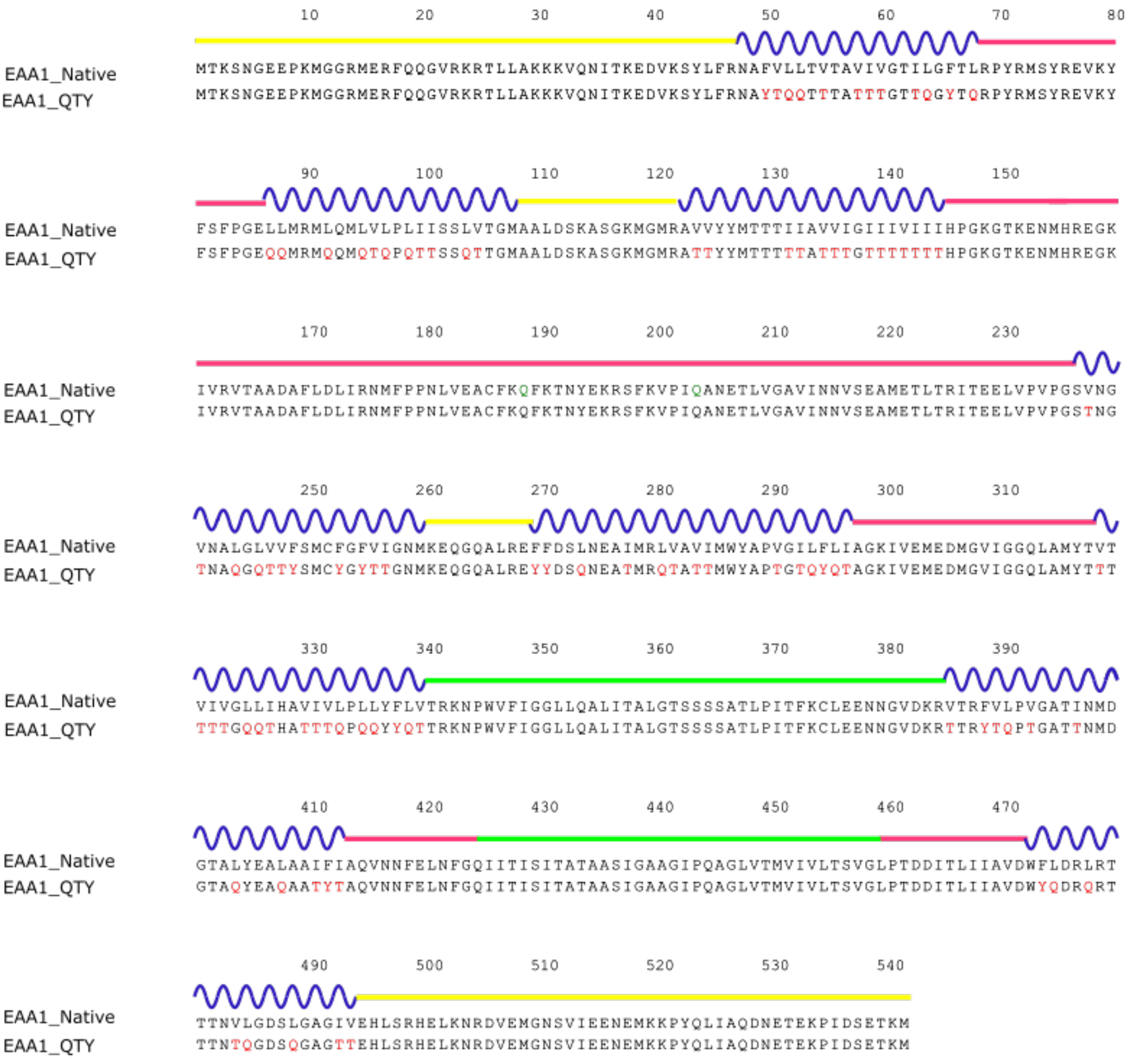

a, EAA1

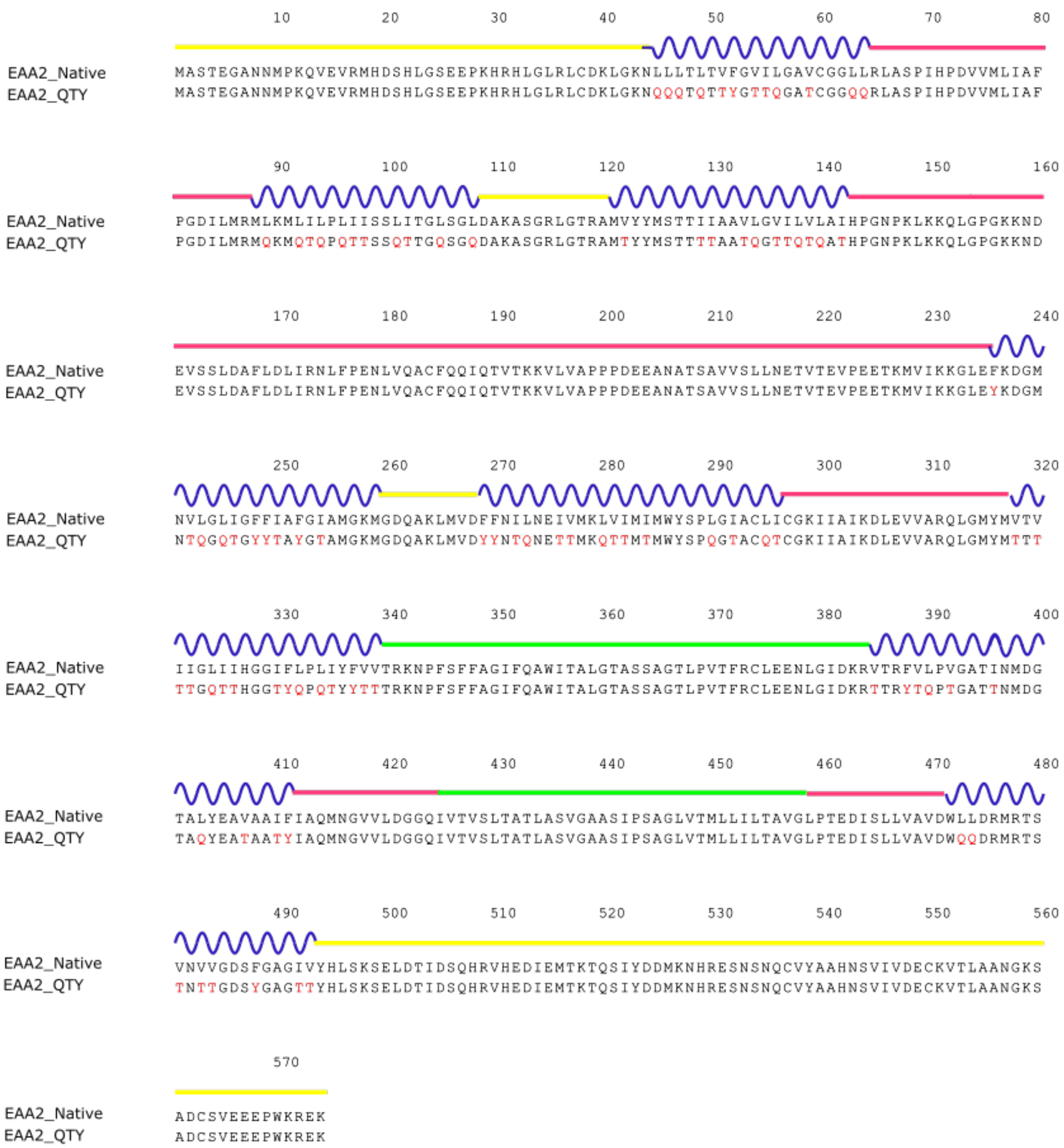
10 20 30 40 50 60 70 80
EAA2_Native MASTEGANNMPKQVEVRMHDSHLGSEEPKHRHLGLRLCDKLGKNLLLTLTVFGVILGAVCGGLLRLASPIHPDVVMLIAF
EAA2_QTY MASTEGANNMPKQVEVRMHDSHLGSEEPKHRHLGLRLCDKLGKNQQQTQTTYGTTQGATCGGQQRLASPIHPDVVMLIAF
90 100 110 120 130 140 150 160
EAA2_Native PGDILMRMLKMLILPLIISSLITGLSGLDAKASGRLGTRAMVYYMSTTIIAAVLGVILVLAIHPGNPKLKKQLGPGKKND
EAA2_QTY PGDILMRMQKMQTQPQTTSSQTTGQSGQDAKASGRLGTRAMTYYMSTTTTAATQGTTQTQATHPGNPKLKKQLGPGKKND
170 180 190 200 210 220 230 240
EAA2_Native EVSSLDAFLDLIRNLFPENLVQACFQQIQTVTKKVLVAPPPDEEANATSAVVSLLNETVTEVPEETKMVIKKGLEFKDGM
EAA2_QTY EVSSLDAFLDLIRNLFPENLVQACFQQIQTVTKKVLVAPPPDEEANATSAVVSLLNETVTEVPEETKMVIKKGLEYKDGM
250 260 270 280 290 300 310 320
EAA2_Native NVLGLIGFFIAFGIAMGKMGDQAKLMVDFFNILNEIVMKLVIMIMWYSPLGIACLICGKIIAIKDLEVVARQLGMYMVTV
EAA2_QTY NTQGQTGYYTAYGTAMGKMGDQAKLMVDYYNTQNETTMKQTTMTMWYSPQGTACQTCGKIIAIKDLEVVARQLGMYMTTT
330 340 350 360 370 380 390 400
EAA2_Native IIGLIIHGGIFLPLIYFVVTRKNPFSFFAGIFQAWITALGTASSAGTLPVTFRCLEENLGIDKRVTRFVLPVGATINMDG
EAA2_QTY TTGQTTHGGTYQPQTYYTTTRKNPFSFFAGIFQAWITALGTASSAGTLPVTFRCLEENLGIDKRTTRYTQPTGATTNMDG
410 420 430 440 450 460 470 480
EAA2_Native TALYEAVAAIFIAQMNGVVLDGGQIVTVSLTATLASVGAASIPSAGLVTMLLILTAVGLPTEDISLLVAVDWLLDRMRTS
EAA2_QTY TAQYEATAATYIAQMNGVVLDGGQIVTVSLTATLASVGAASIPSAGLVTMLLILTAVGLPTEDISLLVAVDWQQDRMRTS
490 500 510 520 530 540 550 560
EAA2_Native VNVVGDSFGAGIVYHLSKSELDTIDSQHRVHEDIEMTKTQSIYDDMKNHRESNSNQCVYAAHNSVIVDECKVTLAANGKS
EAA2_QTY TNTTGDSYGAGTTYHLSKSELDTIDSQHRVHEDIEMTKTQSIYDDMKNHRESNSNQCVYAAHNSVIVDECKVTLAANGKS
570
EAA2_Native ADCSVEEEPWKREK
EAA2_QTY ADCSVEEEPWKREK

b, EAA2

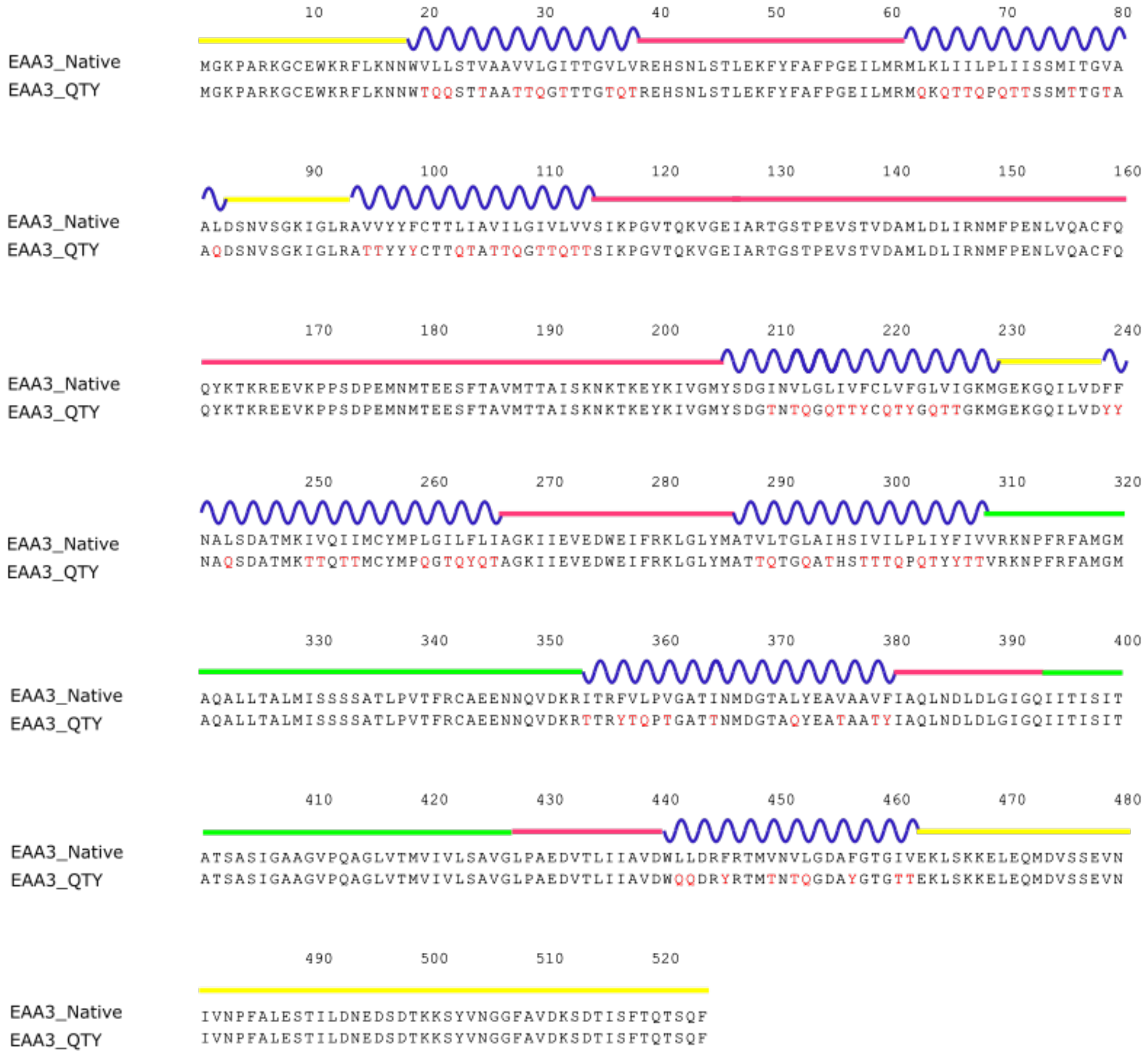

10 20 30 40 50 60 70 80
EAA3_Native MGKPARKGCEWKRFLKNNWVLLSTVAAVVLGITTGVLVREHSNLSTLEKFYFAFPGEILMRMLKLIILPLIISSMITGVA
EAA3_QTY MGKPARKGCEWKRFLKNNWTQQSTTAATTQGTTTGTQTREHSNLSTLEKFYFAFPGEILMRMQKQTTQPQTTSSMTTGTA
90 100 110 120 130 140 150 160
EAA3_Native ALDSNVSGKIGLRAVVYYFCTTLIAVILGIVLVVSIKPGVTQKVGEIARTGSTPEVSTVDAMLDLIRNMFPENLVQACFQ
EAA3_QTY AQDSNVSGKIGLRATTYYYCTTQTATTQGTTQTTSIKPGVTQKVGEIARTGSTPEVSTVDAMLDLIRNMFPENLVQACFQ
170 180 190 200 210 220 230 240
EAA3_Native QYKTKREEVKPPSDPEMNMTEESFTAVMTTAISKNKTKEYKIVGMYSDGINVLGLIVFCLVFGLVIGKMGEKGQILVDFF
EAA3_QTY QYKTKREEVKPPSDPEMNMTEESFTAVMTTAISKNKTKEYKIVGMYSDGTNTQGQTTYCQTYGQTTGKMGEKGQILVDYY
250 260 270 280 290 300 310 320
EAA3_Native NALSDATMKIVQIIMCYMPLGILFLIAGKIIEVEDWEIFRKLGLYMATVLTGLAIHSIVILPLIYFIVVRKNPFRFAMGM
EAA3_QTY NAQSDATMKTTQTTMCYMPQGTQYQTAGKIIEVEDWEIFRKLGLYMATTQTGQATHSTTTQPQTYYTTVRKNPFRFAMGM
330 340 350 360 370 380 390 400
EAA3_Native AQALLTALMISSSSATLPVTFRCAEENNQVDKRITRFVLPVGATINMDGTALYEAVAAVFIAQLNDLDLGIGQIITISIT
EAA3_QTY AQALLTALMISSSSATLPVTFRCAEENNQVDKRTTRYTQPTGATTNMDGTAQYEATAATYIAQLNDLDLGIGQIITISIT
410 420 430 440 450 460 470 480
EAA3_Native ATSASIGAAGVPQAGLVTMVIVLSAVGLPAEDVTLIIAVDWLLDRFRTMVNVLGDAFGTGIVEKLSKKELEQMDVSSEVN
EAA3_QTY ATSASIGAAGVPQAGLVTMVIVLSAVGLPAEDVTLIIAVDWQQDRYRTMTNTQGDAYGTGTTEKLSKKELEQMDVSSEVN
490 500 510 520
EAA3_Native IVNPFALESTILDNEDSDTKKSYVNGGFAVDKSDTISFTQTSQF
EAA3_QTY IVNPFALESTILDNEDSDTKKSYVNGGFAVDKSDTISFTQTSQF

c, EAA3
